# Combined RNA interference and gene replacement therapy targeting *MFN2* as proof of principle for the treatment of Charcot–Marie–Tooth type 2A

**DOI:** 10.1007/s00018-023-05018-w

**Published:** 2023-11-25

**Authors:** Federica Rizzo, Silvia Bono, Marc David Ruepp, Sabrina Salani, Linda Ottoboni, Elena Abati, Valentina Melzi, Chiara Cordiglieri, Serena Pagliarani, Roberta De Gioia, Alessia Anastasia, Michela Taiana, Manuela Garbellini, Simona Lodato, Paolo Kunderfranco, Daniele Cazzato, Daniele Cartelli, Caterina Lonati, Nereo Bresolin, Giacomo Comi, Monica Nizzardo, Stefania Corti

**Affiliations:** 1 Foundation IRCCS Ca’ Granda Ospedale Maggiore Policlinico, Milan, Italy; 2 Dino Ferrari Centre, Neuroscience Section, Department of Pathophysiology and Transplantation (DEPT), University of Milan, Milan, Italy; 3 United Kingdom Dementia Research Institute Centre, Institute of Psychiatry, Psychology and Neuroscience, King’s College London, Maurice Wohl Clinical Neuroscience Institute, London, UK; 4 Istituto Di Genetica Molecolare “Romeo Ed Enrica Invernizzi”, Milan, Italy; 5 Department of Biomedical Sciences, Humanitas University, Pieve Emanuele, 20072 Milan, Italy; 6 IRCCS Humanitas Research Hospital, Rozzano, 20089 Milan, Italy; 7 Fondazione IRCCS Istituto Neurologico Carlo Besta, Milan, Italy; 8 Center for Preclinical Research, Fondazione IRCCS Ca’ Granda Ospedale Maggiore Policlinico, Via Pace 9, 20100 Milan, Italy; 9 Foundation IRCCS Ca’ Granda Ospedale Maggiore Policlinico, Neuromuscular and Rare Diseases Unit, Milan, Italy

**Keywords:** *MFN2*, RNA interfering, Gene therapy, Motor neuron, MitoCharc1, CMT2A

## Abstract

Mitofusin-2 (MFN2) is an outer mitochondrial membrane protein essential for mitochondrial networking in most cells. Autosomal dominant mutations in the *MFN2* gene cause Charcot–Marie–Tooth type 2A disease (CMT2A), a severe and disabling sensory-motor neuropathy that impacts the entire nervous system. Here, we propose a novel therapeutic strategy tailored to correcting the root genetic defect of CMT2A. Though mutant and wild-type *MFN2* mRNA are inhibited by RNA interference (RNAi), the wild-type protein is restored by overexpressing cDNA encoding functional *MFN2* modified to be resistant to RNAi. We tested this strategy in CMT2A patient-specific human induced pluripotent stem cell (iPSC)-differentiated motor neurons (MNs), demonstrating the correct silencing of endogenous *MFN2* and replacement with an exogenous copy of the functional wild-type gene. This approach significantly rescues the CMT2A MN phenotype in vitro, stabilizing the altered axonal mitochondrial distribution and correcting abnormal mitophagic processes. The *MFN2* molecular correction was also properly confirmed in vivo in the MitoCharc1 CMT2A transgenic mouse model after cerebrospinal fluid (CSF) delivery of the constructs into newborn mice using adeno-associated virus 9 (AAV9). Altogether, our data support the feasibility of a combined RNAi and gene therapy strategy for treating the broad spectrum of human diseases associated with *MFN2* mutations.

**Supplementary Information:**

The online version contains supplementary material available at 10.1007/s00018-023-05018-w.

## Introduction

Mitofusin-2 (MFN2) is a highly conserved GTPase anchored to the outer mitochondrial membrane. Together with its homolog Mitofusin 1 (MFN1), MFN2 is involved in regulating the balance between mitochondrial fusion and fission, two processes that are critical in mitochondrial network quality control, cellular stress response, and apoptosis [1–5]. In neurons, MFN2 also plays a tissue-specific role in regulating mitochondrial axonal transport; its dysfunction, which causes energy failure, has been correlated to the pathogenesis of common neurodegenerative diseases, such as Alzheimer’s disease (AD) and Parkinson’s disease (PD) [5–7]. MFN2 is encoded by the nuclear *MFN2* gene, whose mutations are mainly associated with the disease Charcot–Marie–Tooth type 2A (CMT2A; OMIM 609260), the most common subtype of axonal Charcot–Marie–Tooth (CMT) [1, 8–13], but also with severe neurological forms that involve the entire central nervous system (CNS) [3, 13, 14]. CMT2A is clinically characterized by severe neuropathy, predominantly motor, accompanied in some cases by significant proprioception loss [3, 12, 13, 15]. Patients with CMT2A usually begin to experience progressive muscle weakness and atrophy of the legs and arms in childhood, and one-fourth to one-third of them become wheelchair-dependent [3, 10, 12, 13, 15]. In addition to the classic form of CMT2A, a clinical spectrum of uncommon presentations has been reported in some cases, suggesting that MFN2 dysfunction may also lead to CNS and systemic impairment [3, 10, 12, 13, 15]. These phenotypes span from pyramidal and white matter alterations, often in the form of spastic paraparesis, to severe encephalopathies with developmental delay, with a high degree of neurological deficits [15–18]. Additional symptoms may be present, particularly optic nerve atrophy [3, 10, 12–14, 19]. These observations suggest that MFN2 mutations may have a broader impact on the nervous system than previously thought, resulting in a broad spectrum of clinical features and inter-individual variability despite the predominance of a severe phenotype [12]. More than 100 MFN2 mutations have been detected in CMT2A patients, but most of them are missense, whereas a limited number are nonsense variants or deletions. Although a few recessive forms have been described [20–23], CMT2A is generally associated with autosomal dominant or de novo dominant inheritance [8, 9, 12]. MFN2 mutations seem to induce the disease through a “dominant-negative” mechanism, meaning that expression of the wild-type (WT) *MFN2* allele is negatively regulated by the mutant protein [2, 12, 24–26]. These considerations highlight the need to preserve the healthy function of the endogenous protein. No Food and Drug Administration (FDA) or European Medical Agency (EMA)-approved curative or symptomatic medications are currently available, and management is based on supportive care. Thus, the development of meaningful therapeutic strategies for these highly disabling and often fatal mitofusinopathies is urgently needed. A rational approach for slowing or preventing disease progression is based on tackling the underlying genetic cause. Currently, several gene therapy-based strategies are in development for genetic neuromuscular diseases and aim to replace missing or defective genes with the aid of viral vectors [27, 28]. One of the most remarkable therapeutic success is represented by the approval of a gene therapy-based approach for spinal muscular atrophy (SMA), a severe fatal genetic motor neuron disease comprising the delivery of a healthy copy of *SMN* by adeno-associated virus serotype 9 (AAV9) [29]. AAV9 is a relatively safe vector that can effectively transfect neurons and motor neurons (MNs) of the CNS after systemic injection, at least in newborns, or upon cerebrospinal fluid delivery by lumbar puncture at later stages [30], guaranteeing long-term (at least 10 years) genetic correction [31]. Moreover, increasing knowledge on the translational and posttranslational machinery has led to the identification of molecular strategies that are able to down-regulate the gene expression of a toxic gene isoform, such as RNA interference (RNAi) with microRNA or short-hairpin RNA (shRNA) [32]. Although it is still unclear how *MFN2* mutations lead to selective neuronal degeneration, gene therapy that replaces the functional protein while concurrently reducing the mutant one could represent a potentially valuable therapeutic strategy, tackling the root cause of the disease. However, it is necessary to selectively silence the mutant allele, leaving the WT allele intact. This objective is difficult to achieve because the broad spectrum of patient mutations would require a tailored silencing strategy for each or a few patients, with practical difficulties in drug development and regulatory agency approval. Here, we propose a new combined RNAi and gene therapy approach in which both mutant and WT endogenous alleles are inhibited by shRNA, whereas the WT protein is restored using a WT cDNA modified to be resistant to the shRNA. We successfully generated two constructs carrying the shRNA and resistant cDNA. We were then able to achieve effective silencing of the endogenous *MFN2* alleles (both mutant and WT) and replace them with WT shRNA-resistant *MFN2* through in vitro lentiviral delivery in CMT2A MNs from human induced pluripotent stem cells (iPSCs) [33] and in vivo AAV9 delivery into the cerebrospinal fluid in MitoCharc1 mice, a CMT2A model [34]. The expression of WT exogenous MFN2 combined with the silencing of endogenous MFN2 was able to stabilize the mitochondrial distribution and quantity and to rescue abnormal autophagic processes. Our data provide proof-of-concept evidence that the combined RNAi and gene therapy approach may serve as a therapeutic strategy for mitofusinopathies and, potentially, other inherited autosomal dominant neurological diseases.

## Materials and methods

### Cell culture

The iPSC lines from CMT2A patients and healthy subjects were derived from fibroblast samples collected by our institutional biobank with appropriate consent and local ethical committee approval and were already available in our laboratory [33]. The iPSCs were maintained in culture on Matrigel-coated dishes with Essential 8 medium (Thermo Fisher Scientific). Lentiviral transductions, selection and expansion of stable iPSC lines were performed in mTeSR1 (Stem Cell Technologies). HeLa cells were cultured in Dulbecco’s modified Eagle’s medium (DMEM)/F12 supplemented with 15% fetal bovine serum, 1% penicillin/streptomycin (Pen-Strep), and 1% amphotericin B (all from Thermo Fisher Scientific). All cell cultures were maintained at 37 °C in 5% CO_2_. All cell lines were tested for mycoplasma contamination once a month.

### Plasmids

For transient knockdown, pSUPuro-*MFN2*-shRNAtg1 and pSUPuro-*MFN2*-shRNAtg2 were cloned by inserting double-stranded oligos into pSUPERpuro between the BglII and HindIII sites as described previously [35, 36]. The shRNAs expressed from pSUPuro-*MFN2*-shRNAtg1 and pSUPuro-MFN2-shRNAtg2 target nucleotides 1564–1582 (5′-CCTCAAGGTTTATAAGAAT-3′) and 2381–2399 (5-GCAAAGCTGCTCAGGAATA-3′) of *MFN2* mRNA (numbering according to NM_014874.4). pSUPuro-scrambled (5′-ATTCTCCGAACGTGTCACG-3) is described elsewhere [37]. C-terminally Myc-DDK-tagged MFN2 was from Origene (pCMV6-MFN2-Myc-DDK; #RC202218; Myc-tag: EQKLISEEDL  ~ 1202 Daltons; FLAG-tag: DYKDDDDK  ~ 1012 Da).

To render the *MFN2* cDNA RNAi-resistant, silent mutations were introduced into the shRNA target sites in the *MFN2* cDNA using the Quikchange Lightning Multi-Site Kit (Agilent) and primers QC-MFN-t1 (5′-gacttccacccttctccagtagtgctgaaagtctacaaaaacgagctgcaccgccacatagagga-3′) and QC-MFN-t2 (5′-cttgactcacttcagagcaaagctaaactcctgagaaacaaagccggttggttggacagtga-3′) according to the manufacturer’s protocol to generate pCMV6-MFN2Rtg1-Myk-DDK and pCMV6-MFN2Rtg2-Myk-DDK, respectively. For stable knockdown, the shRNAtg2 sequence was inserted into pEco-Lenti-H1-shRNA-Blasticidin (Gentarget). To increase the expression of blasticidin resistance in iPSCs, the RSV promoter between the MluI and KpnI sites of pEco-Lenti-H1-MFN2-shRNAtg2-shRNA-Blasticidin was replaced by a gene-synthesized fragment (GeneArt Gene Synthesis) containing a CMV promoter with a chimeric intron to generate pEco-Lenti-H1-MFN2-shRNAtg2-CMV-BSD. To generate the lentiviral *MFN2* cDNA expression construct (pLVX-EF1a-MFN2Rtg2-Myk-DDK-IRES-Puro), *MFN2* cDNA was excised from pCMV6-MFN2Rtg2-Myk-DDK using EcoRI and PmeI (New England Biolabs) and cloned into the EcoRI-BamHI (Blunt) sites of PLVX-EF1a-IRES-Puro (Clontech). All constructs were verified by Sanger sequencing.

### Transient transfection, lentiviral vector production, and transduction

HeLa cells were transfected with 7.5 µg shRNAtg1 or shRNAtg2 using Lipofectamine LTX reagent (ThermoFisher Scientific) or Dreamfect (OZ Biosciences). Twenty-four hours after transfection, cells were selected in DMEM/F12 medium supplemented with 1.5 µg/mL puromycin (Sigma Aldrich). Co-transfection experiments with 7.5 µg shRNAtg1/MFN2Rtg1 or shRNAtg2/MFN2Rtg2 were performed in parallel in a 1:1 (3.75 µg shRNA to 3.75 µg pCMV6) ratio. After 24 h, cells were cultured in the presence of 500 µg/mL G418 (Sigma Aldrich). Cells were transfected with pSUPuro-SCR under the same conditions as controls. Virus production for protein expression was essentially performed as follows. HEK 293 T cells were transfected with pEco-Lenti-H1-shRNAt2-CMV-BSD supplemented with Lentiviral Packaging plasmid (Gentarget; #HT-Pack) or pLVX-EF1a-MFN2Rtg2-Myc-DDK-IRES-Puro supplemented with Lenti-X HTX packaging mix (Clontech: #631248) according to the manufacturer’s protocol and established methods [36]. Lentiviral supernatants were collected 48 and 72 h post-transfection, filtered through a 0.45-μM polyethersulfone sterile filter (Millipore), followed by concentration using Lenti-X-Concentrator (Clontech: #631232). CMT2A iPSCs were transduced three times with pEco-Lenti-H1-shRNAt2-CMV-BSD supernatant to increase the number of transduced cells without diluting the essential growth factors in the iPSC medium. The cells were incubated twice with lentiviral supernatant overnight. An initial assessment indicated an insufficient MFN2 silencing, so the cells were re-transduced again one month later. Eight hours after the final transduction, cells were expanded under blasticidin selection at a final concentration of 2.5–5 μg/mL, followed by a final 5-day selection with 40 µg/mL blasticidin. To rescue MFN2 expression, pLVX-EF1a-MFN2Rtg2-Myc-DDK-IRES-Puro viral supernatant was added twice to pEco-Lenti-H1-shRNAt2-CMV-BSD transduced cells. Forty-eight hours after the final transduction, cells were expanded with puromycin to a final concentration of 0.5 μg/mL.

### Differentiation of iPSCs into MNs

iPSCs were differentiated into MNs using a multistep protocol modified from Maury et al. [38]. To induce embryoid body (EB) formation from iPSCs, on day 0, iPSCs were dissociated with accutase and resuspended in differentiation N2B27 medium (1:1 DMEM/F12-Neurobasal media, supplemented with N2, B27, 2 mM L-glutamine, 1% Pen-Strep, 0.1 mM β-ME; all from ThermoFisher Scientific), with 10 μM Y-27632 (Cell Signaling Technology), 0.1 μM LDN 193189 (MiltenyiBiotec), 20 μM SB431542, and 3 μM CHIR-99021 (both from Sigma Aldrich). The media was replaced every 2 days, adding small molecules as follows: on day 2, 1 μM LDN 193189 (MiltenyiBiotec), 20 μM SB431542, 3 μM CHIR-99021, and 100 nM retinoic acid (RA, all three from Sigma Aldrich); on day 4: 0.1 μM LDN 193189, 20 μM SB431542, 3 μM CHIR-99021, 100 nM RA, and 500 nM Smoothened Agonist (SAG, Sigma Aldrich); on day 7: 100 nM RA and 500 nM SAG; finally, on day 9: 100 nM RA, 500 nM SAG, and 10 μM DAPT (Stem Cell Technologies). BDNF (20 ng/mL) and GDNF (10 ng/mL; both from Peprotech) were added to the differentiation medium on day 10. On day 11 or 14, EBs were dissociated and the cells seeded on poly-L-ornithine (20 µg/mL) and laminin (20 µg/mL; both from Sigma Aldrich) coated plates. Three or four days after seeding, cells were stained for live imaging analyses, and 5–8 days after seeding, they were fixed for immunocytochemistry and harvested for mtDNA and Western blot analysis.

### Immunocytochemistry of iPSCs and MNs

Cells were fixed in 4% paraformaldehyde for 20 min at 37 °C, permeabilized with 0.25% Triton X-100, and then blocked with 10% bovine serum albumin in phosphate-buffered saline (PBS) containing 0.25% Triton X-100 (all from Sigma Aldrich) for 1 h at room temperature. We incubated the cells with primary antibodies (Table S1) overnight at 4 °C, and then with secondary antibodies (Table S2) for 1 h at room temperature. The nuclei were stained with 0.5 µg/mL DAPI (Sigma Aldrich). Images were acquired using a laser-scanning Leica TCS SP5 confocal microscope (Leica Microsystems, Wetzlar, Germany) or a LED-based Nikon ECLIPSE Ti/CREST microscope (Nikon).

### Mitochondrial area analysis

Accurate analysis of the area occupied by mitochondrial structures requires sharp, high contrast images with minimal background noise. Single maximum intensity projection images of TOM20 immunolabeled MNs obtained by laser-scanning confocal microscopy (Leica TCS SP5, Leica Microsystems) were pre-processed in Fiji software using an unsharp mask filter and then binarized by thresholding (Otsu threshold 20560–65535). To perform single cell analysis of the mitochondrial area, each cell was subdivided into seven adjacent regions of interest (ROIs), the first spanning from the center of the cell to the axon hillock (7 × 10 µm) and the other six covering the axon (3 × 10 µm), for a total length of 70 µm. The mean fluorescence intensity (*I*_average_) was measured.

Quantification of the mitochondrial area was performed according to the specifications of Valente et al. [39]. The mitochondrial area represents the total area in the image covered by signal after being separated from the background and consists of the number of pixels in the binary image containing the signal multiplied by the area of a pixel. *I* represents the pixel intensity in the binarized image, *x* represents the width of the image in pixels, y represents the height of the image in pixels, and s represents the calibrated length of one pixel:

$${\text{Mitochondrial footprint = }}\frac{{I_{{{\text{average}}}} }}{{I_{{{\text{max}}}} }} \cdot x \cdot y \cdot s^{2}$$

Data were graphed and two groups of data were compared by multiple Student’s unpaired *t*-tests using GraphPad Prism 8 (GraphPad Software, San Diego, California, USA). Data were considered to be significantly different if *p* < 0.05.

### Cell imaging of lysosomes and mitochondria

For mitochondrial and lysosome cell imaging, MNs plated in optical 4- or 8-well µ-Slides (Ibidi GmbH) pre-coated with poli-L-ornithine and laminin (both from Sigma Aldrich) were transduced with CellLight™ Mitochondria-RFP BacMam 2.0 and/or CellLight™ lysosome-GFP BacMam 2.0 reagents (both from ThermoFisher Scientific) (30 particles per cell) and incubated for 48 h at 37 °C. Imaging was performed with a Crest Optics Spinning Disk module (Crest-Crisel Instruments) mounted on a fully automated inverted Nikon ECLIPSE Ti microscope (Nikon) and acquisitions achieved with an Andor DU-888 EM-CCD camera (Andor) for fast recordings and NIS-Elements v.5 software (Nikon).

### mtDNA analysis

Total DNA was extracted from MNs using a standard protocol (Flexigene, Qiagen). The mtDNA was quantified by quantitative real-time PCR using the ΔΔCt method on a 7500 Real Time PCR system (Software 2.01, Applied Biosystems, ThermoFisher Scientific) and the Taqman assay with probes for human mitochondrial genes *CYTB* (VIC-CAC CAG ACG CCT CAA CCG CCT T-TAMRA) and *ND4* (FAM-CCG ACA TCA TTA CCG GGT TTT CCT CTT G-MGB), normalizing for nuclear *APP* (FAM-CCC TGA ACT GCA GAT CAC CAA TGT GGT AC-TAMRA) and *RNAseP* (TaqMan™ Copy Number Reference Assay, human, RNase P; 4,403,326) genes, respectively. All quantifications were carried out in triplicate using 25 ng of total DNA as the template. The mtDNA levels were normalized to nuclear DNA and expressed as relative values using the amount of mtDNA in the cells of healthy controls as a reference (relative quantification = 1).

### AAV vectors

AAV9 vectors were produced by Virovek Laboratories (Hayward, CA). Self-complementary AAV9-KD (AAV9-KD) contained an shRNAtg2 sequence (5′-GCAAAGCTGCTCAGGAATA-3′) under the control of the U6 promoter to silence MFN2 (numbering according to NM_014874.4) and incorporated a GFP tag under the control of the CMV promoter. Single-stranded (ss)AAV9-KD-rMFN2 (AAV9-KD-rMFN2) contained an shRNAtg2 sequence (5′-GCAAAGCTGCTCAGGAATA-3′) under the control of the U6 promoter and Myc-DDK-tagged *MFN2* cDNA mutated to be resistant to the shRNA (as previously described) under the control of the CMV promoter. As a control, an AAV9-null vector was produced with a non-coding sequence under the control of the CMV promoter.

### Animal procedures

The MitoCharc1 transgenic mice (B6;D2-Tg(Eno2-MFN2*R94Q)L51Ugfm/J) have the neuron-specific rat enolase (Eno2) promoter directing the expression of human R94Q (arginine to glutamine) MNF2 mainly in neurons, mimicking the most common mutation found in CMT2A patients [34]. Hemizygous mutant mice (MFN2) express two copies of mouse WT murine *mfn2* and one copy of human mutant *MFN2*; they are viable, fertile, normal in size. MFN2 mice and WT littermates were used for the experiments and analyses. The genotypes of the mice were confirmed using a PCR-based assay as described previously [34]. All transgenic animals were purchased from the Jackson Laboratory (stock #012812), and they were maintained according to standard conditions, including ad libitum access to food and water and 12-h dark/light cycle. All animal experiments were approved by the Italian Ministry of Health review boards in compliance with U.S. National Institutes of Health Guide for the Care and Use of Laboratory Animals. AAV9-KD or AAV9-KD-rMFN2 (1.8 × 10^13^ vg/kg) were injected into MitoCharc1 pups (P1, *n* = 3) via intracerebroventricular injection [40, 41]. AAV9::null was used as a control vector (*n* = 3). To evaluate MFN2 expression, brains were collected after 2 weeks.

### Protein analysis

Western blotting was performed as described previously [33, 42]. Protein lysate was separated by 4–12% sodium dodecyl sulfate–polyacrylamide gel electrophoresis (SDS-PAGE) in MES SDS Running Buffer 20x (B0002; life Technologies) for 30 min at 200 V. To separate endoMFN2 and exoMFN2, the gel was ran in MOPSs SDS Running Buffer 20× (B0001; Life Technologies) for 90 min at 130 V and then 30 min at 90 V. Proteins were transferred to a nitrocellulose membrane (GE Healthcare) and incubated with primary antibodies (Table S1) overnight at 4 °C. The membranes were then incubated in secondary antibodies (Table S2) and the immune complexes revealed using the Odyssey® Fc Imaging System (LI-COR Biosciences). Anti-actin antibody was used as a loading control. Semi-quantitative analysis was performed using Image Studio™ Lite software (LI-COR Biosciences).

### Statistical analysis

Statistical analysis was carried out utilizing StatsDirect for Windows (version 2.6.4) or GraphPad Prism 8 software. Multiple comparisons on a single data set were performed with one-way analysis of variance (ANOVA) and, when several variables were considered, two-way ANOVA was used, followed by appropriate post hoc analysis. Two-tailed, unpaired Student’s *t-*test was employed to compare two groups. All experiments were carried out at least in triplicate. The experimental results are shown as mean + SEM or mean + SD as needed. The null hypothesis was rejected at the 0.05 level of significance.

## Results

### Combined RNAi/gene replacement therapy effectively promotes silencing of endogenous MFN2 and restoration of exogenous wild-type MFN2 protein levels in CMT2A cells

**Fig. 1:**
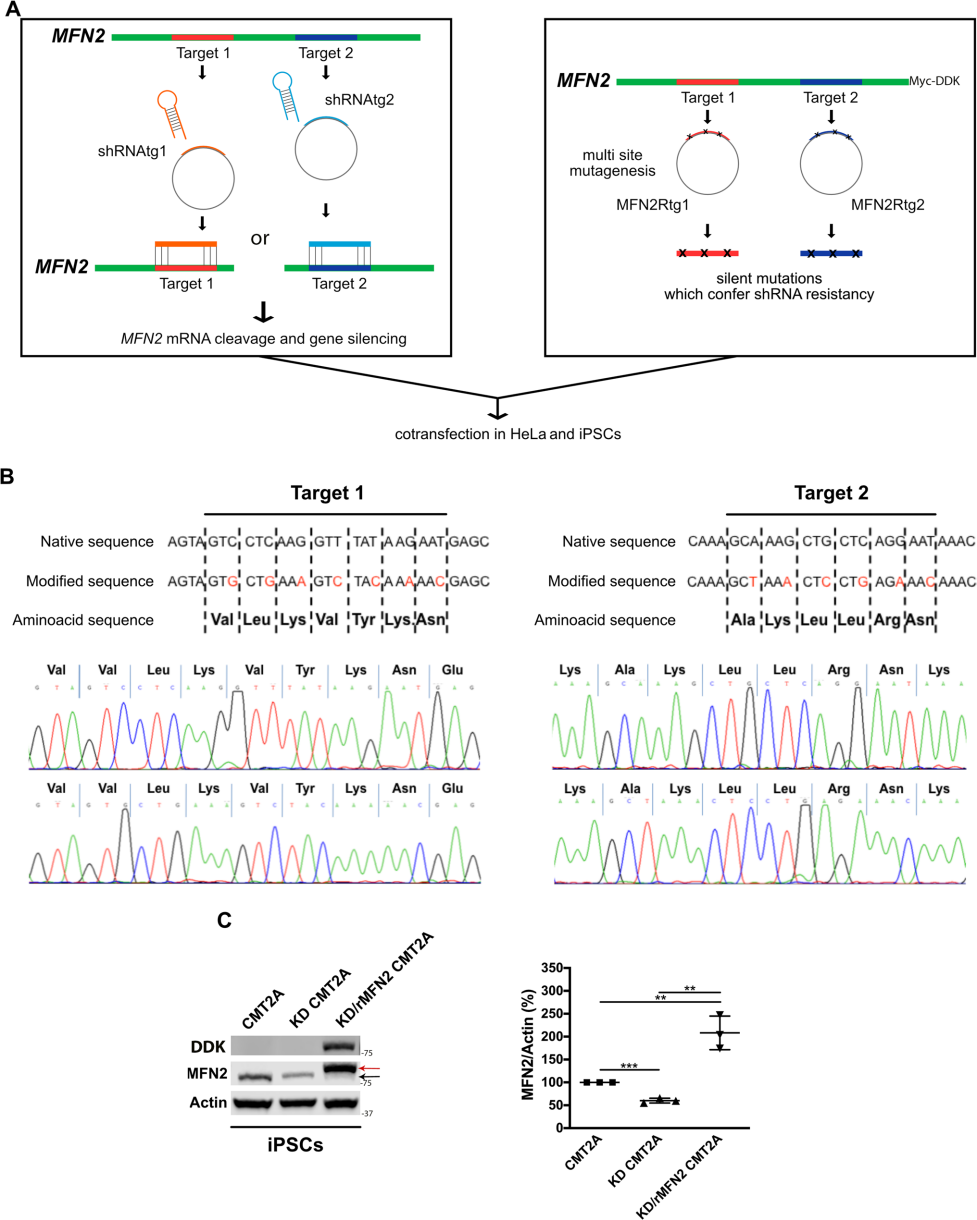
CMT2A iPSCs showed silencing of endogenous* MFN2* and overexpression of modified exogenous *MFN2* after combined RNAi/gene therapy.** A** Schematic representation of *MFN2* gene silencing and *MFN2* multi-site mutagenesis experiments in CMT2A iPSCs and HeLa cells. **B** Diagram of multi-site mutagenesis performed on target1 and target2 of human *MFN2*, respectively. Mutated nucleotides are in red. There was no change in the amino acid sequences between native and mutated sequences. Direct sequencing of vector-mutated sequences confirmed the presence of inserted mutations. **C** Representative Western blot of endogenous MFN2 (endoMFN2, black arrow) and RNAi-resistant exogenous WT Myc-DDK MFN2 (exoMFN2, red arrow) in CMT2A iPSCs (CMT2A) after shRNAtg2 transduction (KD-CMT2A) or after shRNAtg2 and MFN2Rtg2 co-transduction (KD/rMFN2-CMT2A). The specific expression of exogenous MFN2 (Myc-DDK MFN2) was also confirmed by Western blot using an antibody for DYKDDDDK Tag (DDK). Densitometric quantification (n = 3). Error bars indicate SEM of MFN2/Actin expression. **P < 0.01, ***P < 0.001, Student’s *t*-test

To develop a therapeutic strategy for CMT2A, we investigated a combined approach of RNA interference and gene replacement therapy (RNAi/gene therapy) in which both *MFN2* mutant and WT alleles are inhibited by a shRNA sequence, while the Myc-DDK-tagged WT MFN2 protein is restored leveraging the transfer of a Myc-DDK-tagged *MFN2* cDNA engineered to be resistant to the shRNA (Fig. 1A). We identified and synthesized two shRNA sequences targeting *MFN2* (shRNAtg1 and shRNAtg2) on a genomic stretch free of known CMT2A mutations. In parallel, we modified Myc-DDK-tagged WT *MFN2* cDNA to be resistant to shRNAtg1 (RNAi-resistant MFN2 tg1, MFN2Rtg1) or shRNAtg2 (RNAi-resistant MFN2 tg2, MFN2Rtg2), inserting silent mutations (Fig. 1B). Thanks to this strategy, the modified resistant cDNA escapes silencing from designed shRNA and translates into the WT MFN2 protein. Correct mutations in the construct were verified through direct Sanger sequencing of the plasmids (Fig. 1B). The vectors were tested in vitro by co-transfecting HeLa cells with either shRNAtg1/MFN2Rtg1 or shRNAtg2/MFN2Rtg2. Western blot analysis showed a reduction in the endogenous MFN2 (endoMFN2; *P* < 0.0001) protein levels and overexpression of exogenous Myc-DDK-tagged WT MFN2 (exoMFN2; *P* < 0.0001) protein using both constructs, though the most efficient combination was shRNAtg2/MFN2Rtg2 (Fig. S1). Based on these results, only target2 was used in subsequent experiments. Patient-specific CMT2A iPSCs were transduced with lentiviral vectors either encoding shRNAtg2 or shRNAtg2 and respective RNAi-resistant MFN2 (MFN2Rtg2). Cells treated with shRNAtg2 will hereafter be defined as KD, cells treated with MFNRtg2 as rMFN2, and cells treated with both shRNAtg2 and MFNRtg2 as KD/rMFN2. Western blotting confirmed silencing of the endoMFN2 protein in iPSCs (*P* < 0.001) and the restoration of exoMFN2 levels (*P* < 0.01; Fig. 1C).

**Fig. 2:**
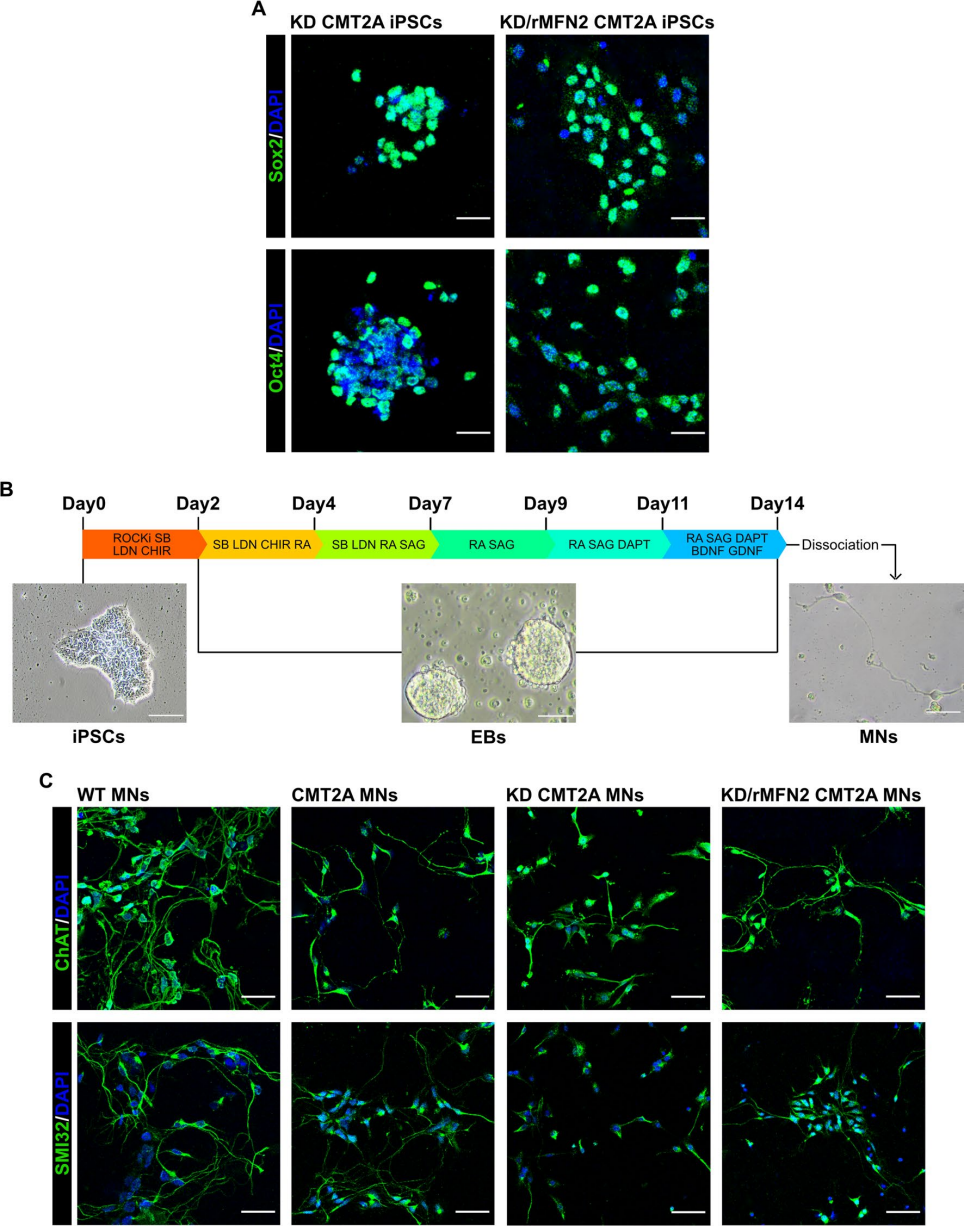
Combined RNAi/gene therapy preserved iPSC and MN phenotypes.** A** Immunocytochemistry of KD-CMT2A and KD/rMFN2-CMT2A iPSCs for pluripotency transcription factors SOX2 (green) and OCT4 (green). Nuclei were labeled with DAPI (blue). Images were acquired using a Leica TCS SP5 confocal microscope at 20 ×  magnification. Scale bar: 25 μm. **B** Experimental outline for MN differentiation of WT, CMT2A, KD-CMT2A, and KD/rMFN2-CMT2A iPSCs and bright-field representative images at three different differentiation time points (iPSC, EB, and MN). Scale bars: 100 μm for iPSCs, 50 μm for both EBs and MNs. **C** Immunocytochemistry of WT, CMT2A, KD-CMT2A, and KD/rMFN2-CMT2A MNs after 2 weeks of differentiation for MN markers SMI32 (green) and ChAT (green). Images were acquired using a Leica TCS SP5 confocal microscope at 20 × magnification. Nuclei were labeled with DAPI (blue). Scale bar: 50 µm

After RNAi/gene therapy, stable iPSC lines maintained their embryonic stem cell morphology and expression of pluripotency markers (Fig. 2A).

### Combined RNAi/gene replacement therapy increases mitochondrial content and normalizes mitochondrial distribution in CMT2A MNs

**Fig. 3:**
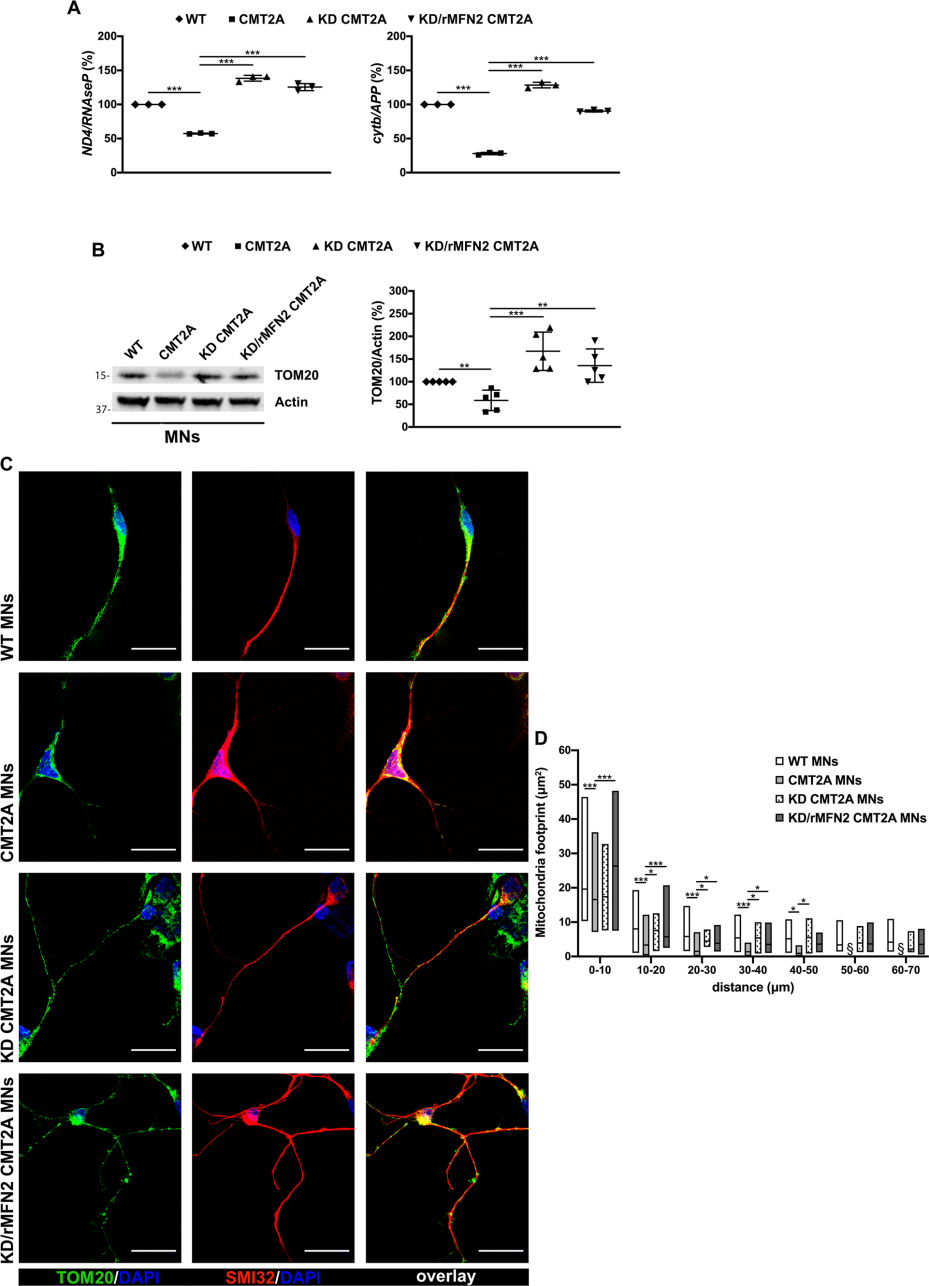
Reduced mitochondrial content and altered mitochondrial distributions were rescued after combined RNAi/gene therapy in CMT2A MNs. **A** Quantitative PCR analysis of human mitochondrial genes (*ND4* and *cytb*) and nuclear genes (*RNAseP* and *APP*) in WT, CMT2A, KD-CMT2A, and KD/rMFN2-CMT2A MNs. Error bars indicate SEM of *ND4/RNAseP* and *cytb/APP*: ***P < 0.001, one-way ANOVA. **B** Representative Western blot analysis of the mitochondrial protein TOM20 in WT, CMT2A, KD-CMT2A, and KD/rMFN2-CMT2A MNs. Error bars indicate SEM of TOM20/actin expression. **P < 0.01, ***P < 0.001, one-way ANOVA. **C** Representative images of WT, CMT2A, KD-CMT2A, and KD/rMFN2-CMT2A MNs labeled with TOM20 (red) and SMI32 (green). Images were acquired using a Leica TCS SP5 confocal microscope at 63 × magnification. Nuclei were labeled with DAPI (blue). Scale bar: 25 µm. **D** Quantification of the mitochondrial area (µm^2^) in the four different MN cultures. Box plots show the median (horizontal lines) and the minimum and maximum (box) values. Multiple Student’s unpaired t-test; *P < 0.05, ***P < 0.001 from 0 to 50 µm; § undetectable fluorescence; *n* = 30 for WT-MNs, *n* = 48 for CMT2A MNs, *n* = 13 for CMT2A-KD MNs, and *n* = 45 for KD/rMFN2-CMT2A MNs in two independent experiments

As MNs are selectively affected in CMT2A pathogenesis, we evaluated the efficacy of our RNAi/gene therapy strategy in patient-specific CMT2A iPSC-derived MNs, analyzing key MN features relevant to the disease that have already been identified as being impaired [33]. To obtain spinal MNs, control (WT), CMT2A, or stable-engineered KD and KD/rMFN2 iPSC lines were differentiated using a multistep protocol (Fig. 2B) modified from Maury et al. [38]. After 2 weeks of differentiation, cells expressed ChAT and SMI32, indicating MN maturation with no significant differences between engineered and non-engineered cells (Figs. 2C, S2). As demonstrated previously [33], we did not observe differences in survival and axonal elongation between WT and CMT2A MNs (Fig. S3). Similarly, the silencing of endoMFN2 and expression of resistant exo MFN2 did not modify these characteristics (Fig. S3). We also previously reported that MNs derived from CMT2A patients exhibit a global reduction in mitochondrial content and altered mitochondrial distribution [33], in line with data from patients and murine models [2, 25, 43]. When assessing these features in our stable-engineered lines, we observed an increase in mitochondrial content in both KD and KD/rMFN2-CMT2A MNs compared to CMT2A, as demonstrated by the mitochondrial DNA (mtDNA) quantity analysis (*P* < 0.001; Fig. 3A) and TOM20 protein expression (*P* < 0.01; Fig. 3B). Abnormal axonal mitochondrial trafficking is implicated in the pathogenesis of several subtypes of axonal CMT. Neurons expressing mutant MFN2 exhibit altered localization of mitochondria due to their slower anterograde/retrograde movements along axons [2, 25, 43–47]. Our group already observed that mitochondria tend to cluster around the nucleus in CMT2A MNs rather than to spread homogeneously along the axons [33], suggesting that abnormal mitochondrial trafficking may be part of the pathophysiology of CMT2A. Performing mitochondrial area analysis for the mitochondrial protein TOM20 signal, we confirmed a significantly progressive reduction in the amount of mitochondria, which is indicative of mitochondrial distribution, in adjacent regions along the whole axon length of CMT2A cultures compared to controls (*P* < 0.05; Fig. 3C, D; Table S3). Notably, our RNAi/gene therapy strategy restored a normal mitochondrial distribution (*P* < 0.05; Fig. 3C, D; Table S3).

Following RNAi/gene therapy, CMT2A MNs had a global increase in mitochondrial content and normalization of the altered mitochondrial distribution, supporting the idea that our strategy could have therapeutic relevance for human CMT2A.

### Combined RNAi/gene replacement therapy reduces mitophagy in CMT2A MNs

**Fig. 4:**
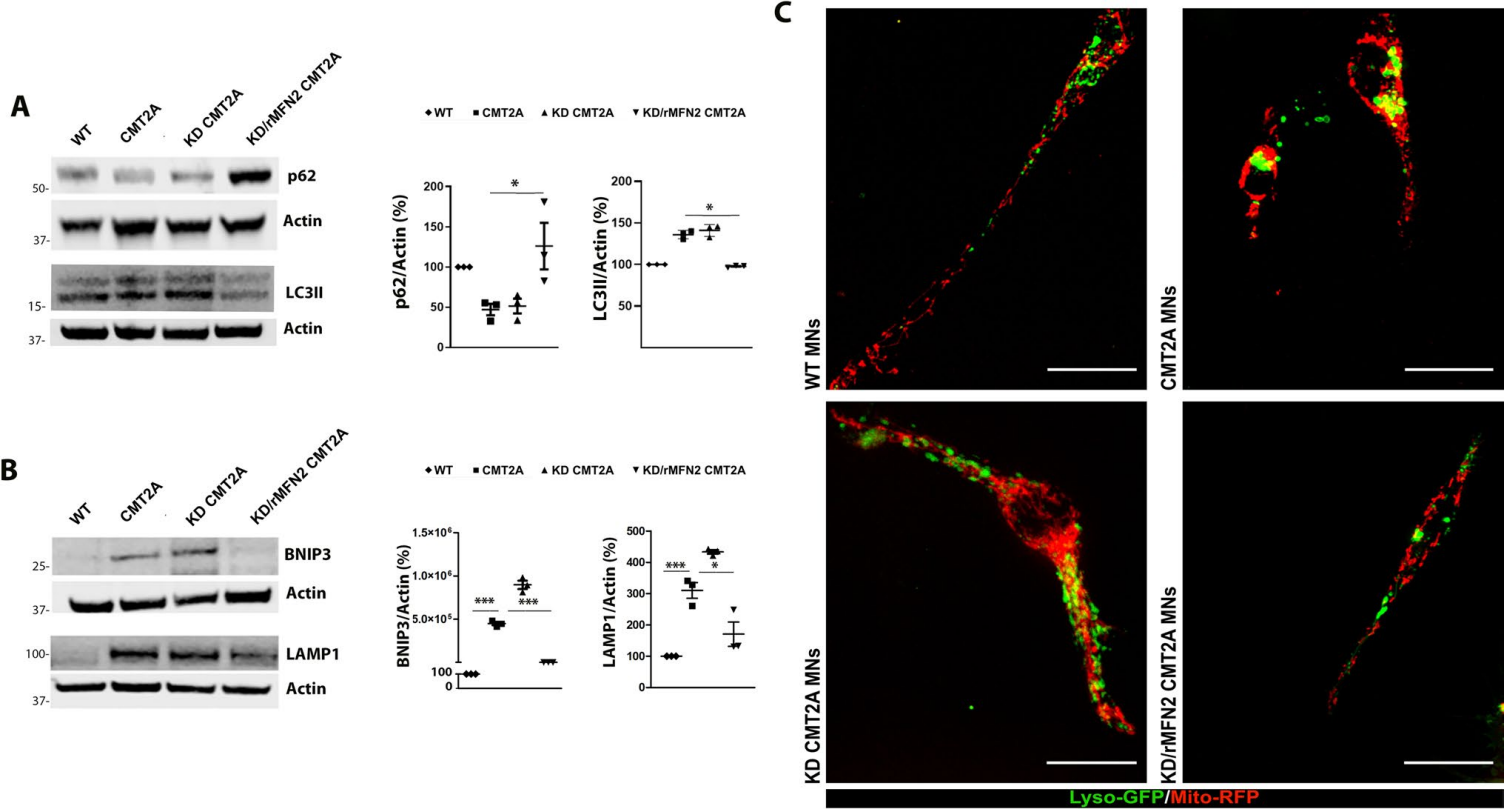
Combined RNAi/gene therapy reduced the impairment in the mitophagic flux in CMT2A MNs. **A** Western blot analysis of p62 and LC3-II in WT, CMT2A, KD/rMFN2-CMT2A, and KD-CMT2A MNs. Error bars indicate SEM of p62/actin and LC3-II/actin expression. *P < 0.05, one-way ANOVA. **B** Western blot analysis of BNIP3 and LAMP1 in WT, CMT2A, KD/rMFN2-CMT2A, and KD-CMT2A MNs. Error bars indicate SEM of BNIP3/actin and LAMP1/actin expression. ***P < 0.0001, *P < 0.05, one-way ANOVA. **C** Lysosome (green) and mitochondria (red) localization in WT, CMT2A, KD/rMFN2-CMT2A, and KD-CMT2A MNs. The yellow signal indicates the overlapping of lysosomes and mitochondria. Images were acquired using a Nikon ECLIPSE Ti/CREST microscope at 60 × magnification with spinning disk. Scale bar: 25 µm. Two independent experiments included *n* = 11 for WT-MNs, *n* = 10 for CMT2A MNs, *n* = 9 for CMT2A-KD MNs, and *n* = 8 for KD/rMFN2-CMT2A MNs

According to our previously published RNA sequencing and protein study, *MFN2* mutations likely induce an increased autophagic flux and mitochondrial clearance in CMT2A MNs [33], altering the balance between mitochondrial biogenesis and the regulation of autophagy and lysosomal activity. During autophagy initiation, autophagosome formation is associated with the conversion of cytosolic-associated protein light chain 3 (LC3-I) to the membrane-bound LC3-II form, LC3 then binds to the adaptor protein p62/SQSTM sequestrome which facilitates the autophagic degradation of ubiquitinated protein aggregates in lysosomes. Thus, p62 and LC3 are routinely used as biomarkers to monitor the level of autophagy [48]. Therefore, to monitor the effect of RNAi/gene therapy on general autophagy, the expressions of autophagy markers p62 and LC3-II were evaluated in KD and KD/rMFN2-CMT2A MNs compared the untreated CMT2A MNs (Fig. 4A). A reduction in the autophagic substrate p62 and an increase in the LC3-II expression in CMT2A MNs confirmed the activation of autophagy in CMT2A MNs. Notably, after combined RNAi/gene therapy autophagy was reduced (*P* < 0.05; Fig. 4A), whereas silencing per se did not show a significant effect (*P* < 0.05; Fig. 4A). Beside the factors involved in general autophagy (p62 and LC3-II), mitophagy is characterized by the induction of proteins that accumulate on mitochondria before their removal such as BNIP3, which plays a critical role in altering the permeability and dysfunction of mitochondria and thus enhance mitophagy [49]. Interestingly, the levels of BNIP3 were increased in CMT2A MNs compared to WT-MNs (*P* < 0.001; Fig. 4B), confirming the activation of mitophagy in CMT2A cells. Notably, the levels of BNIP3 were reduced after RNAi/gene therapy (*P* < 0.001; Fig. 4B), but no therapeutic effects were observed with *MFN2* silencing alone. The activation of the mitophagic flux was confirmed by an increase in lysosomal-associated membrane protein 1 (LAMP1) expression (*P* < 0.001; Fig. 4B). Restorative effects were detected with combined RNAi/gene replacement therapy (*P* < 0.05; Fig. 4B), but not with *MFN2* silencing alone. The dysfunctional distribution and overlap of lysosomes with mitochondria around the nucleus in CMT2A MNs compared to WT-MNs confirmed the alteration of general autophagy and selective mitophagy. This parameter was reverted upon RNAi/gene therapy (Fig. 4C).

Overall, combined RNAi/gene therapy was able to restore sensitivity to reduce mitophagy in human CMT2A cells, supporting the idea that this strategy could have beneficial effects on the CMT2A disease phenotype.

### Combined administration of RNAi/gene replacement therapy by AAV9 results in silencing of endogenous MFN2 and expression of exogenous MFN2 in the CNS of MitoCharc1 mice

**Fig. 5:**
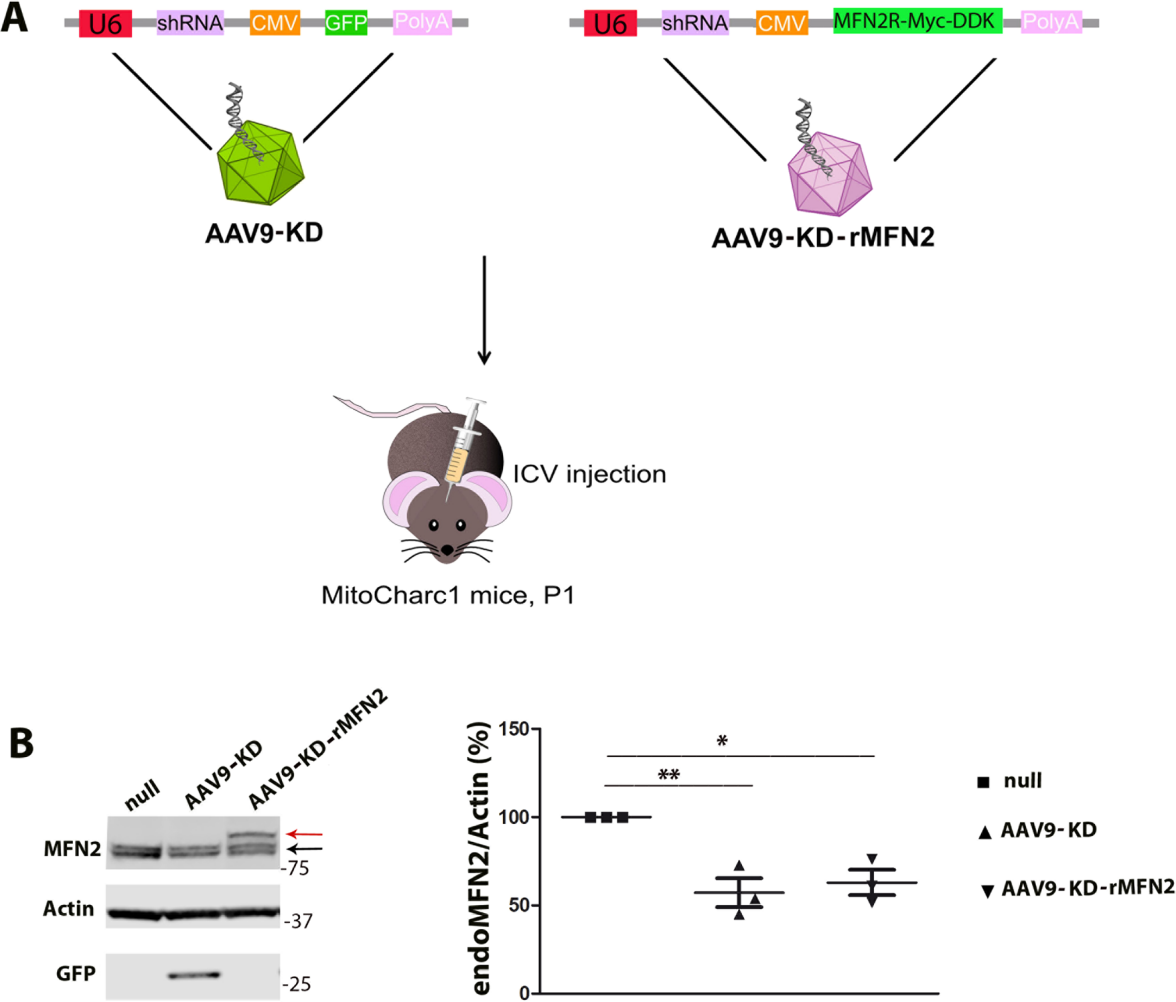
AAV9-KD and AAV9-KD-rMFN2 efficiently transduced tissues in CMT2A mice. **A** In vivo experimental design. Newborn (P1) CMT2A were injected intracerebroventricularly (ICV) with AAV9-KD or AAV9-KD-rMFN2. **B** Representative Western blot of endogenous MFN2 protein (endoMFN2, black arrow) and exogenous MFN2 (Myc-DDK MFN2, shift of higher molecular weight, red arrow) after AAV9-KD, AAV9-KD-rMFN2 or AAV9::null treatment in the brains of MitoCharc1 mice. AAV9-KD transduction in the brains was confirmed by the detection of GFP tag. Densitometric quantification (*n* = 3). Error bars indicate SEM of endoMFN2/actin expression. **P < 0.01,*P < 0.05, Student’s *t*-test

To allow in vivo assessment of our RNAi/gene therapy as CMT2A therapy, we evaluated our approach in a CMT2A mouse model. We used MitoCharc1, a transgenic mouse model expressing the mutant human MFN2-R94Q under the control of the neuron-specific rat promoter enolase 2 (*Eno2*) [34], the only model available when this study started. Considering that AAV9 displays CNS tropism and crosses the blood–brain barrier (BBB) after systemic and intrathecal administration [50], and that AAV9–mediated gene therapies have been developed for neurological diseases [40], we selected AAV9 as a shuttle for in vivo administration of our strategy. More specifically, we produced two AAV9 vectors, one containing shRNAtg2 with a GFP tag (AAV9-KD) to silence the endogenous MFN2 and one carrying both shRNAtg2 and Myc-DDK-tagged MFN2 cDNA mutated at the genomic level to be resistant to shRNA (AAV9-KD-rMFN2) to silence endogenous MFN2 and re-express WT MFN2 at the same time. To evaluate the molecular effect after AAV9 administration, we treated a cohort of MitoCharc1 mice at P1 with a single intracerebroventricular injection of AAV9-KD-rMFN2 or AAV9-KD (Fig. 5A). The analysis of MFN2 expression showed significant silencing of endogenous MFN2 in the brains of AAV9-KD-rMFN2 (*P* < 0.001) or AAV9-KD (*P* < 0.01) treated animals at the protein level compared to AAV9-null treated animals. The overexpression of exogenous MFN2 was shown by a distinct band on Western blot at a higher molecular weight (red arrow, Fig. 5B). These data confirm the molecular efficacy of our strategy in vivo. Next, we analyzed the effects of our strategy on the pathological phenotype in MitoCharc1 mice. This mouse model has already been shown to display a mild phenotype with impairment in gaiting parameters and endurance starting at 5 months of age [34, 51]. However, in our experimental setting, MitoCharc1 did not manifest an overt neuromuscular pathological phenotype (Figs. S5, S6, S7). We further characterized these transgenic animals in terms of both neuromotor (Fig. S5) and peripheral sensory phenotypes (Fig. S7) to search for any other biomarkers that may be altered. More specifically, we monitored not only weight and survival (Fig. S5), but also motor coordination (Rotarod test, Fig. S6), pain response (hot plate test, Fig. S6), and neuropathological (neuromuscular junction analysis, Fig. S6; dorsal ganglia immunophenotyping characterization, Fig. S7) and neurophysiological features (motor and sensory nerve conduction studies, Figs. S6, S7). Overall, we did not observe any significant differences between MFN2 and WT animals in our transgenic colony at 5 months of age and later.

In parallel, we evaluated the presence of potential transcriptomic alterations as disease biomarkers, performing total RNA-sequencing analysis on lumbar spinal cords from MFN2 mutant animals compared to WT animals (Fig. S8A). Despite the transcriptomic analyses disclosing 1119 differentially expressed genes in MFN2 animals versus WT animals (Table S4), explaining a significant separation between the two genotypes in the principal component analysis (Fig. S8B), no relevant MFN2-related pathways emerged when implementing gene set enrichment analysis (GSEA; Table S5). Therefore, we could not identify significant pathological hallmarks to be used as outcome measures of our therapy, rendering it difficult to use this transgenic model to convincingly validate any therapeutic effectiveness of our strategy.

Overall, the data on MFN2 expression in the brains of treated Mitocharc1 mice confirmed the molecular efficacy of our strategy in vivo.

## Discussion

CMT2A is a severe debilitating neurological disease that currently lacks a curative treatment. Though how MFN2 mutations lead to selective neuronal degeneration is not yet known, gene therapy may represent a potential curative strategy because it tackles the root cause of the genetic defect. In contrast to recessively inherited diseases, the treatment of autosomal dominant disorders, such as CMT2A, requires the dominant allele to be silenced without altering expression of the WT gene. From this perspective, RNAi mediated by small RNAs, including shRNA, is a powerful gene knockdown technique that allows controlled suppression of the mutant gene [32]. As shRNA targets a specific gene sequence, the modulation of shRNA specificity for a mutant allele with minimal suppression of the corresponding WT alleles is theoretically feasible as a gene therapy technique [52]. Viral delivery of mutant allele-specific shRNAs has been effective in several disease models, including Alzheimer’s disease [53], Parkinson’s disease [54], Huntington’s disease [55, 56], Machado–Joseph disease [57], and amyotrophic lateral sclerosis (ALS) [58]. However, although shRNAs could theoretically discriminate single nucleotide alterations, designing such specific shRNAs is challenging and would require the production and validation of a different construct for each human mutation. To overcome this problem, we investigated a combined approach of global MFN2 RNAi and gene therapy in which transcripts from both endogenous *MFN2* mutant and WT alleles are inhibited by an shRNA sequence that targets a shared mutation-free *MFN2* mRNA sequence while the WT exogenous protein is restored by overexpression of MFN2 cDNA engineered to be resistant to the shRNA, upon insertion of silent mutations. Notably, to develop this combined approach, we screened two shRNA sequences targeting *MFN2* (shRNAtg1 and shRNAtg2) and their corresponding WT *MFN2* cDNA to be resistant to shRNAtg1 (MFN2Rtg1) or shRNAtg2 (MFN2Rtg2). Although shRNAtg1 silenced endoMFN2 better than shRNAtg2, it also silenced exoMFN2 after MFN2Rtg1 co-transfection. Therefore, the shRNAtg2/MFN2Rtg2 combination seemed to be the most efficient one to silence endoMFN2 while maintaining high levels of exoMFN2. Though mild overexpression of exoMFN2 obtained in vitro with target1 can be enough to revert the pathological phenotype, the complexity of the in vivo system could require more robust exoMFN2 expression to obtain therapeutic effects for clinical translation.

In this study, we tested the strategy both in in vitro and in vivo disease models, providing proof of principle of its feasibility. Regarding the in vitro model, combined RNAi/gene therapy induced silencing of the endogenous MFN2 and restored exogenous modified MFN2 levels in CMT2A iPSCs, demonstrating the molecular feasibility of our approach. After RNAi/gene therapy, we observed restoration of the mitochondrial content, as demonstrated by mtDNA quantity analysis and TOM20 protein level expression, and the normalization of altered mitochondrial distribution in CMT2A MNs. Considering the pivotal role of mitochondria in the different pathways that regulate cellular function and survival, this rescue could support the overall energy metabolism of MNs and promote their resilience to degeneration. MNs are long-lived cells that persist throughout the lifespan of the individual and, as such, are more susceptible to the accumulating damage arising from mitochondrial dysfunction. Therefore, the preservation of a healthy pool of correctly localized mitochondria is essential for MN survival, justifying the association of mitochondrial dysfunction with a large number of neurodegenerative disorders [7, 59, 60]. In addition to modulating mitochondrial dysfunction, our combined therapy normalized general autophagy and specific mitochondrial clearance in human CMT2A cells, reverting phenotypic and molecular dysfunction in a human cellular model and supporting the idea that this strategy could be effective for human disease. However, the optimal balance of MFN2 expression levels required for MN homeostasis should be further elucidated for clinical applications. Interestingly, we observed that autophagy and mitophagy were rescued in vitro only when MFN2 expression was restored and not only upon silencing of aberrant MFN2. On the other hand, the mitochondrial distribution was improved (despite not completely) with MFN2 silencing alone, suggesting that also partial reduction of MFN2 may help alleviate the dominant-negative effects and improve cellular functions. Thus, the WT MFN2 could play a more active role in the autophagy/mitophagy pathway than in the regulation of mitochondrial distribution; therefore, silencing both toxic and WTMFN2 can affect its role in autophagy/mitophagy (loss of function), whereas the downregulation of aberrant MFN2 protein, is enough to rescue mitochondrial distribution. Nonetheless, considering all the in vitro data, we cannot conclusively determine whether the MFN2 silencing alone or the MFN2 overexpression alone are able to rescue the overall pathological phenotype, but further studies should be performed to disentangle this aspect. As CMT2A is an autosomal dominant disease with a possible toxic gain-of-function of the protein, silencing alone deserves investigation. On the other hand, testing the extent to which MFN2 overexpression alone, independent of the knockdown, is effective is not irrelevant considering that dominant-negative activities can be out-competed by overexpression of wild-type protein as already demonstrated by MFN1 overexpression which can protect against mitochondrial fusion and transport defects caused by MFN2 mutants in vitro [11, 46] and in vivo [61] models.

To assess the clinical translatability of this therapeutic approach, we tested its molecular efficacy in an in vivo disease model. We selected the viral vector AAV9 [50], which exhibits CNS tropism and crosses the BBB after systemic and intrathecal administration [62]. AAVs are safe and non-pathogenic and have been used in more than 50 clinical trials of human gene therapy (www.clinicaltrials.gov). Furthermore, significant progress has been made in the use of AAV9 gene therapy for the treatment of neurodegenerative diseases. Recently, AAV9-based gene therapy AAV9-SMN was approved by the FDA and EMA to treat SMA patients. Moreover, enrollment in a phase 3 clinical trial on gene therapy for SMARD1 with AAV9-IGHMBP2 vector, which we contributed to at the preclinical level [41], has now entered the recruitment phase (NCT05152823, www.clinicaltrials.gov). Leveraging this scientific progress, we generated two AAV9 vectors, one vector containing the *shRNAtg2* (AAV9-KD) to silence endogenous MFN2 and the other containing both *shRNAtg2* and a single-stranded copy of *MFN2Rtg2* mutated to be resistant to shRNAtg2 (AAV9-KD-rMFN2) to be tested in the MitoCharc1 mouse model [34]. We chose to treat newborn affected mice intracerebroventricularly, injection comparable with the intrathecal one in humans [62, 63]. Indeed, gene transfer to neonates has several advantages over treatment of the adults enabling prevention of onset of the genetic disease, allowing good biodistribution through CNS and CSF, reducing costs and risk of immune rejection [50, 64]. In addition to that, MFN2 was shown to be especially important during developmental stages, therefore, an early treatment would increase the chances of success [1, 10, 65]. The administration of AAV9-KD-rMFN2 resulted in the satisfactory silencing of endogenous MFN2 and overexpression of exogenous MFN2 in the CNS of MitoCharc1 mice, in accordance with the data obtained by AAV9 gene therapy for other neurodegenerative diseases [41, 62, 64]. These data confirm the efficacy of our strategy at the molecular level in vivo in our experimental setting.

In addition to the molecular validation, it is also important to evaluate the therapeutic efficacy of this approach in an animal model of disease to observe the modification of disease biomarkers. Only a few mouse models reproducing CMT2A have been generated, and the ability of these models to recapitulate the disease phenotype is limited [51], making them less reliable for a convincing validation of the therapeutic effectiveness of a therapeutic strategy. The MitoCharc1 transgenic mouse is certainly the most well-known models and harbors the neuron-specific rat *Eno2* promoter driving the expression of human R94Q (arginine to glutamine) MFN2 in neurons, mimicking the most common *MFN2* mutation in CMT2A patients [34]. These mice were already described as having a very mild phenotype but, in our experimental setting, MitoCharc1 did not present an overt neuromuscular disease in functional tests. The identification in these mice of a handy clinical and pathological hallmark that can be used as outcome measure is crucial to clinical translation. Since alterations of a subcellular biomarker, even if present, might not be relevant enough to be suitable for use as a therapeutic clinical outcome, we investigated motor and sensory parameters and performed neuropathological analysis on both neuromuscular junctions and the DRGs. Unfortunately, no significant alterations were observed in MFN2 animals compared to control ones. In addition, we also evaluated potential transcriptomic deregulation in presymptomatic MitoCharc1 mice, considering that altered gene expression related to a pathological phenotype should be detectable before the appearance of the clinical pathological phenotype. Despite the presence of transcriptional differences, no relevant MFN2-related pathways clearly emerged from GSEA. Overall, the lack of a clear pathological phenotype in the mouse model hindered the possibility of demonstrating the therapeutic efficacy of our strategy, making this transgenic model difficult to use.

Despite isolated cases of therapies that moved directly to a clinical trial without the need for testing in animal models, validation of the therapeutic efficacy in animals with a marked pathological phenotype remains an essential step for gene modulation and replacement techniques. Recently, a novel transgenic mouse line expressing MFN2-R94Q under the neuronal-specific Thy1.2 promoter was developed and appears to show evident disease phenotypes [61]. Interestingly, this new model could represent a realistic disease model to validate our proposed strategy, monitoring also any eventual toxic effects, as the next step of our study.

Aside from these considerations, our results suggest the feasibility of a combined RNAi and gene therapy strategy as a potential therapeutic approach for treating the broad spectrum of human diseases associated with MFN2 mutations. We expect that this strategy could determine a shift in the paradigm of CMT2A treatment, as this is the first CMT2A gene therapy able to revert phenotypic and molecular dysfunction in a human cellular model, supporting the idea that this strategy could be effective as a treatment for the human disease. Although some strategies, such as treatment with mitofusin agonists (MAs) [47] or pharmacological histone deacetylase (HDAC6) inhibition [66] and overexpression of MFN1 have obtained promising results in in vitro and in vivo models [61], no effective therapy for CMT2A has yet been successfully translated from the bench to the bedside. Our therapeutic strategy could be decisive because it is aimed at correcting the genetic defect, also considering the promising data obtained with similar AAV9 strategies in other MN diseases.

However, as mentioned above, though this proof-of-concept *MFN2* RNAi/gene therapy approach was able to revert the disease phenotype in human cells, a therapeutic approach in patients requires consideration of several additional points. First, shRNA target sequences must be carefully screened for undesired off-target effects and modified accordingly [67]. Second, though MFN2 overexpression did not result in any overt undesirable toxic phenotype in vivo, long-term monitoring for safety reasons is required, as overexpression toxicity has been reported for other gene replacement therapies [68–70]. Therefore, the development and use of promoters for cDNA expression that mimic endogenous promoter activity or are identical to the endogenous promoter sequence constructs without exceeding AAV packaging capacity is highly desirable [71].

Nonetheless, our proof-of-concept study points out the suitability of a combined approach based on concomitant gene modulation and replacement for the treatment of inherited autosomal dominant neurological diseases, such as CMT2A. Proving the effectiveness in vitro and in vivo, our data pave the way for future preclinical and clinical trials.

### Supplementary Information

Below is the link to the electronic supplementary material.

Supplementary file1 (XLSX 1423 KB)

Supplementary file2 (XLS 11705 KB)

Supplementary file3 (XLSX 2013 KB)

Supplementary file4 (DOCX 1214 KB)

## Data Availability

All relevant data are in the manuscript and supplementary information. The dataset generated and analyzed during the current study are available in the GEO repository (GSE201264).

## Abbreviations

AD: Alzheimer’s disease
PD: Parkinson’s disease
SMA: spinal muscular atrophy
FDA: Food and Drug Administration or
EMA: European Medical Agency
ICV: intracerebroventricularly injection
MFN2: Mitofusin-2
CMT2A: Charcot–Marie–Tooth type 2A disease
RNAi: RNA interference
iPSC: Induced pluripotent stem cell
MN: Motor neuron
CSF: Cerebrospinal fluid
AAV9: Adeno-associated virus 9
CNS: Central nervous system
shRNA: Short-hairpin RNA
endoMFN2: Endogenous MFN2
exoMFN2: Exogenous Myc-DDK-tagged WT MFN2
AAV9-KD: AAV9 containing shRNAtg2 with a GFP tag
AAV9-KD-rMFN2: AAV9 containing shRNAtg2 and Myc-DDK-tagged WT MFN2
MFN2 mice: MitoCharc1 transgenic mice
WT mice: C57BL/6 mice
P: Post-natal day
