# Supplementary material for: Combined RNA interference and gene replacement therapy targeting MFN2 as proof of principle for the treatment of Charcot–Marie–Tooth type 2A
Source: Cell Mol Life Sci. 2023 Nov 25;80(12):373. doi: 10.1007/s00018-023-05018-w (PMC10676309; doi:10.1007/s00018-023-05018-w)
Supplement: Supplementary file 4 — Supplementary file4 (DOCX 1214 KB) [file 18_2023_5018_MOESM4_ESM.docx]

**Supplementary Material and Methods**

**Survival and axon length analysis**

The DeadEnd^TM^ Fluorometric Tunel System protocol (Promega) was used to evaluate cellular survival. The TUNEL positive cells were counted on the total of DAPI positive cells (3 wells/condition/experiment in three experiments). Axonal length studies were performed by measuring soma diameter and length distance to the most distal point of the axon (3 wells/condition/experiment in three experiments). Differences were analyzed using Fiji ImageJ software. All quantifications were performed blind to the genetic status of the material and the treatment. For all imaging, we used a confocal LEICA LCS8.

**Mouse behavioral analysis**

Survival and weight were monitored in MFN2 (n=26) versus WT (n=22) mice from 1 to 24 months. The animals were sacrificed at 24 months.

**Rotarod test**. Motor functions of MFN2 (n=9) and WT (n=10) mice were tested once a month by an accelerating test on a rotarod device (4 to 40 rpm, Rota-Rod 7650, UgoBasile Biological Instruments, Varese, Italy).

**Hot plate test.** MFN2 (n=9, P270) and WT mice (n=10, P270) were placed on a hot plate analgesiometer (UgoBasile Biological Instruments, Varese, Italy) with the surface temperature maintained at 50±0.2°C. The latency before the first licking or lifting of the posterior paw was recorded as the withdrawal latency time. Immediately after the initial reaction, the trial was halted and the mouse removed from the hotplate. To avoid any injury to the tissues, if the animal was not responsive, the test was stopped using a cut-off time of 45 s. Each animal was tested twice with a 30 min interval between the sessions. All tests were performed blinded to the mouse genotype.

**Nerve conduction velocity**

Tail motor and sensory conductions were studied in MFN2 mice (n=8, P270) versus WT mice (n=3, P270) [1]. Briefly, the tail sensory nerve was stimulated by inserting the cathode 1 cm from the tail tip and anode 0.5 cm distally using a square-wave pulse of 0.1-ms duration at supramaximal intensity. Sensory nerve action potentials were recorded by inserting an active electrode 4 cm proximally from the cathode and the reference electrode 0.5 cm more proximally. The sensory nerve conduction velocity was calculated by measuring the latency to the peak of the initial negative deflection and the distance between stimulating and recording electrodes. Motor tail nerve conduction was measured using a bipolar recording configuration. An active electrode was placed 1 cm from the tail tip and reference electrode 0.5 cm distally. The motor tail nerve was stimulated first 2.5 cm proximally from the active recording electrode and then 5 cm proximally. Motor nerve conduction velocity was calculated by subtracting the distal from the proximal compound motor action potential’s first negative peak latency measured in milliseconds, and the difference was divided by the distance between the two stimulating electrodes in millimeters. All of the neurophysiological determinations were performed under standard conditions in a temperature-controlled room; animals were maintained under deep isoflurane anaesthesia during the recordings.

**DRG culture**

Adult DRG neurons were rapidly dissected on ice-cold DMEM (ThermoFisher Scientific) and centrifuged for 3 min at 300 g. They were digested for 20 min in 2.5% collagenase in Ca^2+^- and Mg^2+^-free Hank’s Buffer Saline Solution (HBSS, GIBCO, ThermoFisher Scientific) at 37°C. Additional digestion was carried out for 20 min in 0.25% trypsin (ThermoFisher Scientific) and stopped by washing in DMEM (GIBCO, Life Technologies) containing 15% serum (GIBCO, ThermoFisher Scientific). DRG were dissociated with a pipette and centrifuged at 300 g before re-suspension in DMEM containing 15% FBS and counted. Cells were then plated on 12-mm-diameter glass coverslips in 24-well plates previously coated with Matrigel and left to adhere for 24 hours. The following day, the medium was replaced with neurobasal medium (GIBCO, ThermoFisher Scientific) supplemented with B27, 2 mM L-glutamine, and 1% Pen-Strep (all from ThermoFisher Scientific), and the cells were left to spread for 3-4 days before immunocytochemical analyses.

**Immunohistochemical analysis of murine tissues**

MFN2 (n=6) and WT mice (n=6) were euthanized at P270. Excised tissues (quadriceps, tibial anterior, and DRG) were fixed in 4% paraformaldehyde for 24 h, soaked in 20% sucrose solution overnight, and then frozen in liquid nitrogen-cooled isopentane [2]. Frozen tissues were cryosectioned (20 µm) and mounted on gelatinized glass slides. One every ten sections was collected and analyzed. All sections were saturated with 10% bovine serum albumin and 0.3% Triton X-100 for 1 h at room temperature before incubation with primary antibodies overnight at 4°C (Table S1). The next day, slides were incubated with Alexa Fluor secondary antibodies (Table S2). A Leica TCS SP5 confocal microscope (Leica Microsystems) or Nikon ECLIPSE Ti/CREST microscope (Nikon) were used to acquire images.

**RNA isolation and quantitative RT-PCR**

Total RNA was extracted from the lumbar spinal cords of MFN2 (n=8, P30) and WT mice (n=4, P30) using the RNeasy Mini Kit (Qiagen). Concentrations were measured on a Nanodrop spectrophotometer. Only samples with ratios between 1.8 and 2.0 were further analyzed. A reverse-transcribed 1 μg of total RNA for each sample using the Ready-To-Go kit (GE Healthcare).

**RNA extraction and 3′-mRNA sequencing**

Total RNA was extracted from the lumbar spinal cords of MFN2 (n=8, P30) and WT mice (n=4, P30) using the RNeasy Mini Kit (Qiagen). Concentrations were measured on a Nanodrop spectrophotometer. Only samples with ratios between 1.8 and 2.0 were further analyzed. We reverse-transcribed 1 μg of total RNA for each sample using the Ready-To-Go kit (GE Healthcare). Total RNA extracted from samples was subjected to poly(A) mRNA sequencing. Libraries were constructed using the SMARTer-Stranded Total RNA Kit (Clontech) according to the manufacturer’s instructions. Sequencing was performed on a NextSeq 500 (Illumina). All libraries were sequenced in paired-end mode (75-bp length).

**mRNA sequencing analysis**

Raw reads were preprocessed for adapter trimming. Quality was assessed using the FastQC tool (http://www.bioinformatics.babraham.ac.uk/projects/fastqc). Reads were aligned to the reference genome (Ensembl*Mus musculus* release GRC38) using the STAR algorithm [3]. Differential expression analysis was performed using the Generalized Linear Model approach implemented in the R/Bioconductor edgeR[4] package (R version 3.5; edgeR version 3.24.3) using an FDR ≤ 0.05. The fold change ranked gene list was subjected to GSEA Preranked [5], using the c2.all.v7.5.1 and c5.all.v7.5.1 gene sets with classical enrichment statistics and phenotype permutation.

**Supplementary figure and table**

**Supplementary Table 1.** Primary Antibodies used for immunohistochemistry (IHC), immunocytochemistry (ICC), and Western blotting (WB).

| **Primary Antibodies** | | | | | | |
| --- | --- | --- | --- | --- | --- | --- |
| **Target** | **Manufacturer** | **Host species** | | **Dilution** | | |
|  | | | | **IHC** | **ICC** | **WB** |
| α-BTX, AF-555 | ThermoFisher Scientific (#B35451) | |  | 1:200 |  |  |
| Actin | Sigma Aldrich (#A2066) | | Rabbit |  |  | 1:1000 |
| ATF3 | Abcam (#ab180842) | | Rabbit |  | 1:200 |  |
| β-III tubulin | Biolegend (#801202) | | Mouse |  | 1:1500 |  |
| β-III tubulin | Abcam (#ab18207) | | Rabbit |  | 1:200 |  |
| BNIP3 | Abcam (#ab109362) | | Rabbit |  |  | 1:1000 |
| ChAT | Chemicon (#AB144P) | | Goat |  | 1:250 |  |
| DYKDDDDK Tag | Cell signaling (#2368) | | Rabbit |  |  | 1:1000 |
| DYKDDDDK Tag | Bethyl (#A190-101) | | Goat |  |  | 1:1000 |
| GFP | Invitrogen (#A21311) | | Rabbit |  |  | 1:2000 |
| LAMP1 | Abcam (#ab24170) | | Rabbit |  |  | 1:1000 |
| LC3 I/II | Millipore (ABC929) | | Rabbit |  |  | 1:500 |
| MAP2 | Sigma Aldrich (#M4403) | | Mouse |  | 1:100 |  |
| MFN2 | Cell Signaling (#11925) | | Rabbit |  |  | 1:1000 |
| OCT4 | ThermoFisher Scientific (#701756) | | Rabbit |  | 1:250 |  |
| OLIG2 | Sigma Aldrich (#SAB1404798) | | Mouse |  | 1:25 |  |
| SMI-32 | Abcam (#ab28029) | | Mouse | 1:1000 | 1:600 |  |
| SMI-32 | Abcam (#ab8135) | | Rabbit |  | 1:600 |  |
| SOX2 | Abcam (#ab97959) | | Rabbit |  | 1:500 |  |
| SQSTM1 (p62) | Millipore (#MABN130) | | Mouse |  |  | 1:1000 |
| TOM-20 | Sigma Aldrich (#HPA011562) | | Rabbit | 1:50 | 1:50 | 1:1500 |

**Supplementary Table 2.** Secondary Antibodies used for immunohistochemistry (IHC), immunocytochemistry (ICC), and Western blotting (WB).

| **Secondary Antibodies** | | | | |
| --- | --- | --- | --- | --- |
|  |  | **Manufacturer** | **Dilution** | |
|  | |  | **IHC/ICC** | **WB** |
| Goat anti-Rabbit IgG (H+L) Highly Cross-Adsorbed Secondary Antibody, Alexa Fluor 488 | | ThermoFisher Scientific (#A-11034) | 1:1000 |  |
| Goat anti-Mouse IgG (H+L) Highly Cross-Adsorbed Secondary Antibody, Alexa Fluor 488 | | ThermoFisher Scientific (#A-11029) | 1:1000 |  |
| Goat anti-Rabbit IgG (H+L) Cross-Adsorbed Secondary Antibody, Alexa Fluor 568 | | ThermoFisher Scientific (#A-11011) | 1:1000 |  |
| Goat anti-Mouse IgG (H+L) Cross-Adsorbed Secondary Antibody, Alexa Fluor 568 | | ThermoFisher Scientific (#A-11004) | 1:1000 |  |
| Rabbit anti-Goat IgG (whole molecule)-FITC | | Sigma Aldrich (#F7367) | 1:400 |  |
| Donkey anti-rabbit IRDye 680R | | LI-COR (926-68073) |  | 1:20000 |
| Donkey anti-mouse IRDye 680RD | | LI-COR 926-68072 |  | 1:20000 |
| Donkey anti-rabbit IRDye 800CW | | LI-COR (926-32213) |  | 1:20000 |
| Donkey anti-mouse IRDye 800CW | | LI-COR (926-32212) |  | 1:20000 |
| Donkey anti mouse IRDye 680LT | | Li-COR (926-68022) |  | 1:10000 |
| Donkey anti Goat IRDye 800CW | | LI-COR (926-32214) |  | 1:10000 |

**Supplementary Table 3.** Quantification of the mitochondrial area in one representative cell for WT, CMT2A, KD and KD/rMFN2-CMT2A cultures (related to figure 3D).

**Supplementary Table 4.** List of genes with significantly altered expression in lumbar spinal cord of Mitocharc1 mice (MFN2) compared to control mice (WT).

**Supplementary Table 5.** GSEA of pathways using ranked gene loadings in Mitocharc1 mice (MFN2) *versus* control mice (WT).

**
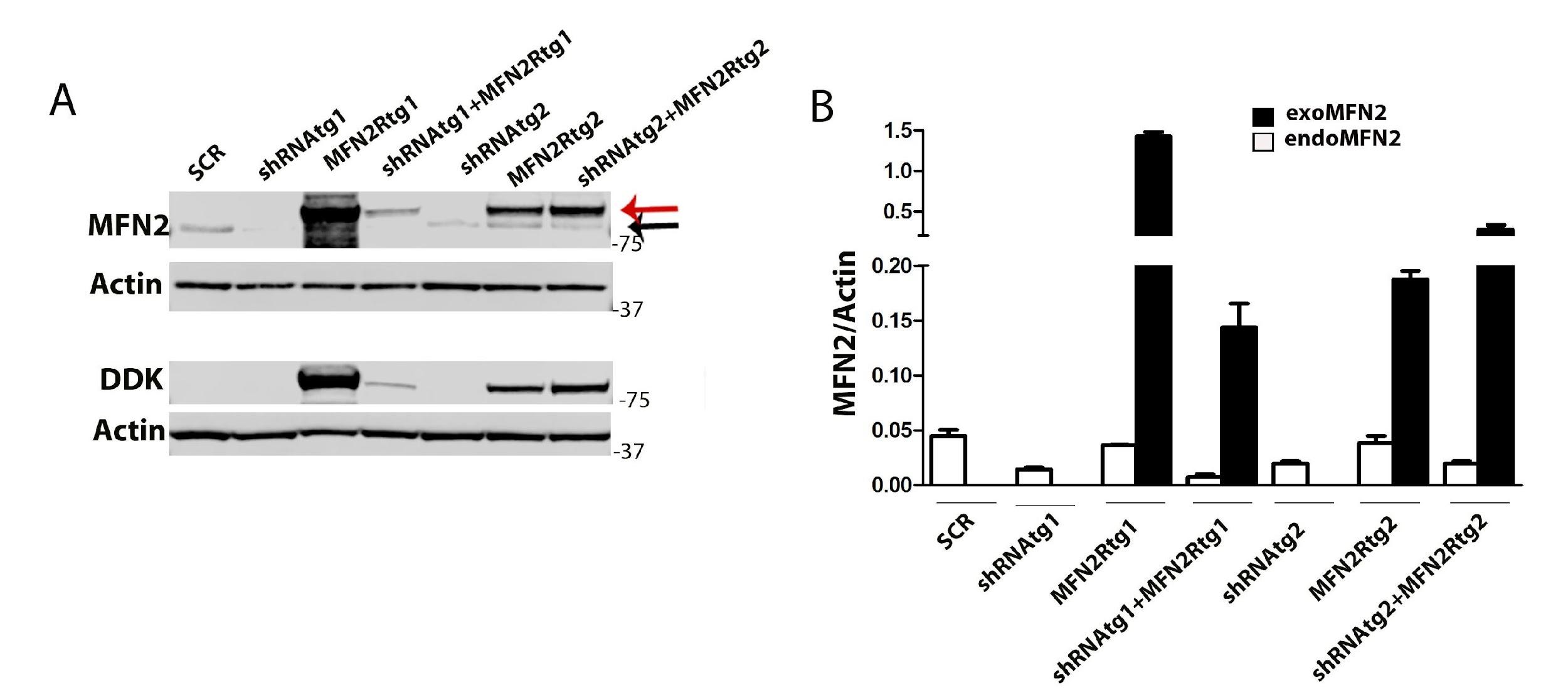
**

**Supplementary Fig. 1. Silencing of endogenous *MFN2* and overexpression of resistant exogenous *MFN2* in HeLa cells after RNAi/gene therapy. A** Representative western blot of endogenous MFN2 (endoMFN2, black arrow; lower molecular weight) and exogenous Myc-DDK WT MFN2 (exoMFN2, red arrow; higher molecular weight) after transfection with shRNAtg1 or shRNAtg2, MFN2Rtg1 or MFN2Rtg2 and shRNAtg1/MFN2Rtg1 or shRNAtg2/MFN2Rtg2 in HeLa cells compared to scramble (SCR) cells. The specific expression of Myc-DDK WT MFN2 was also confirmed using an antibody to DYKDDDDK Tag (DDK). **B** Densitometric quantification (n=3) of endoMFN2 (****P* < 0.0001, One way ANOVA) and exoMFN2 (****P* < 0.0001, One way ANOVA). Error bars indicate SEM of endoMFN2 or exoMFN2/actin expression levels.

**
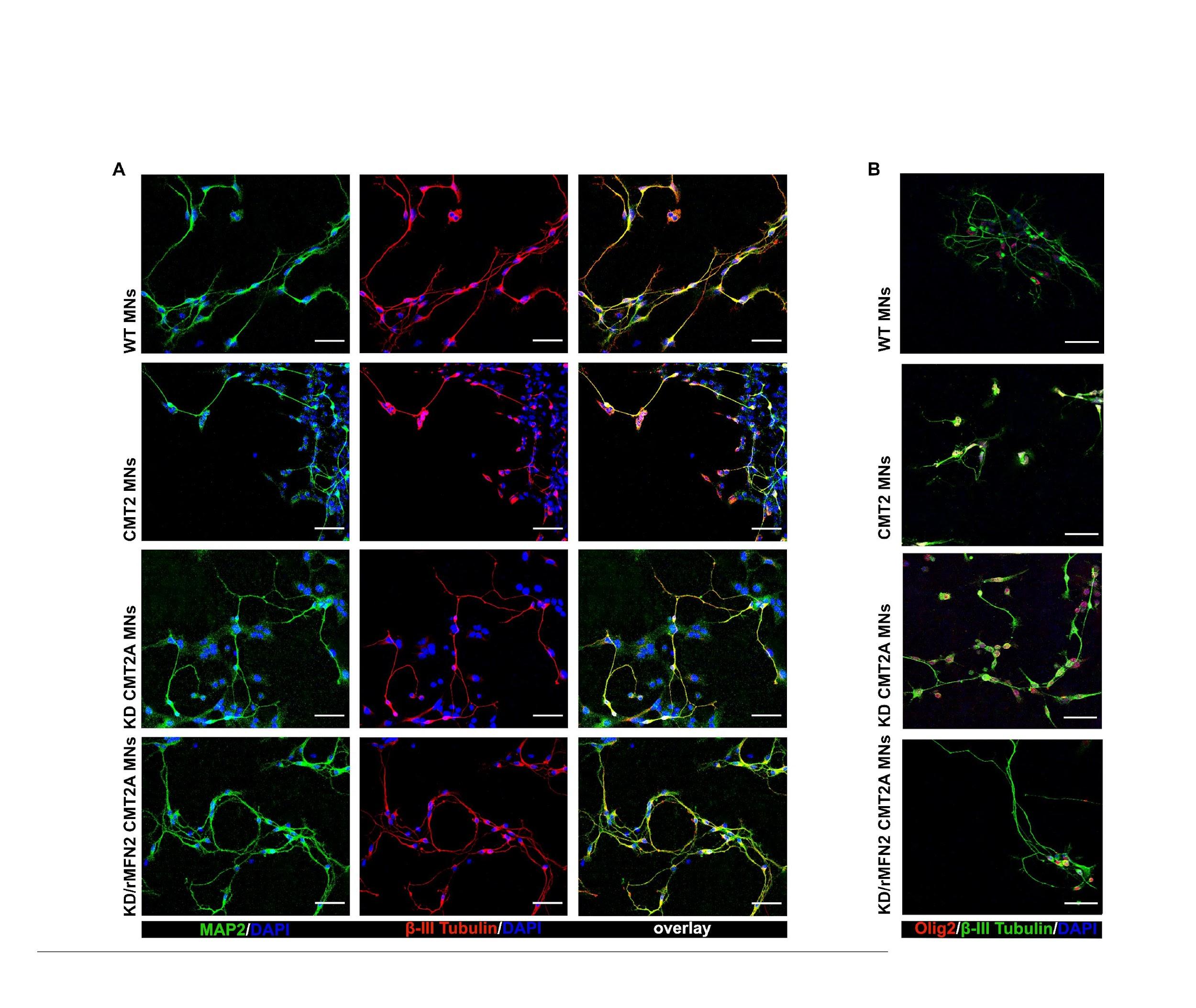
**

**Supplementary Fig. 2. Expression of neuronal markers after iPSC differentiation.** Representative confocal images of WT, CMT2A, KD-CMT2A and KD/rMFN2 MNs: **A** MAP2 (green), βIII-Tubulin (red); **B** OLIG2 (red) and βIII-Tubulin (green). Nuclei were labeled with DAPI (blue). Images were acquired using a Leica TCS SP5 confocal microscope (Leica Microsystems), with a 20x objective. Scale bars: 50 µm.


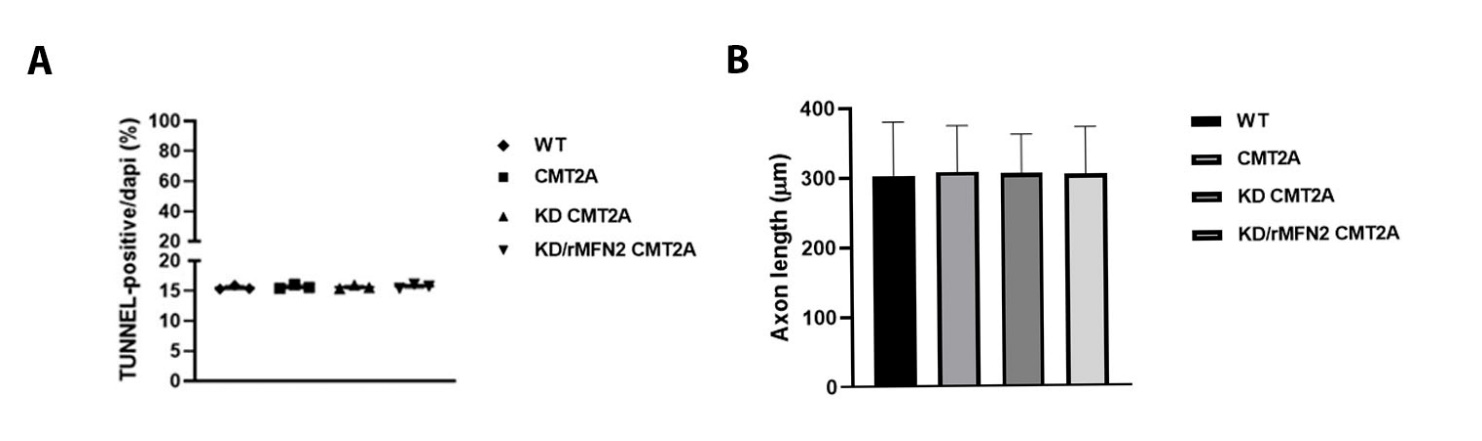


**Supplementary Fig. 3. CMT2A, KD-CMT2A and KD/rMFN2 MNs did not exhibit survival and axon length defects.** **A** Quantification of tunnel signal in WT, CMT2A, KD-CMT2A and KD/rMFN2 MNs (3 wells/condition/experiment in three experiments; *P=*0,8542, One-way ANOVA). **B** Axon length (µm) of WT, CMT2A, KD-CMT2A and KD/rMFN2 MNs (3 wells/condition/experiment in three experiments; *P=*0,9789, One-way ANOVA). Data are represented as mean ± SEM.

**
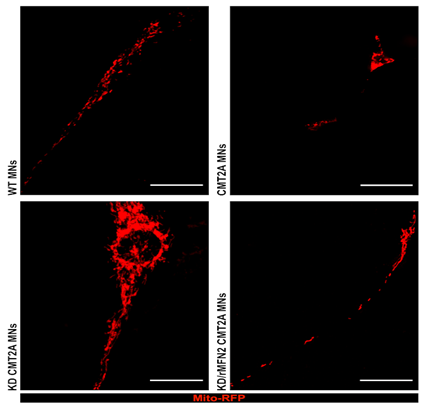

Supplementary Fig. 4. Mitochondria cellular distribution (related to Figure 3)**. **A** Representative z-stack from high-resolution confocal images of mitochondria in WT, CMT2A, KD-CMT2A, and KD/rMFN2 MNs labeled using CellLight™ Mitochondria-RFP, BacMam 2.0 technology (red). Images were acquired using a Nikon ECLIPSE Ti/CREST microscope (Nikon), at 60x magnification, equipped with spinning disk. Scale bar: 25 µm.

**A**


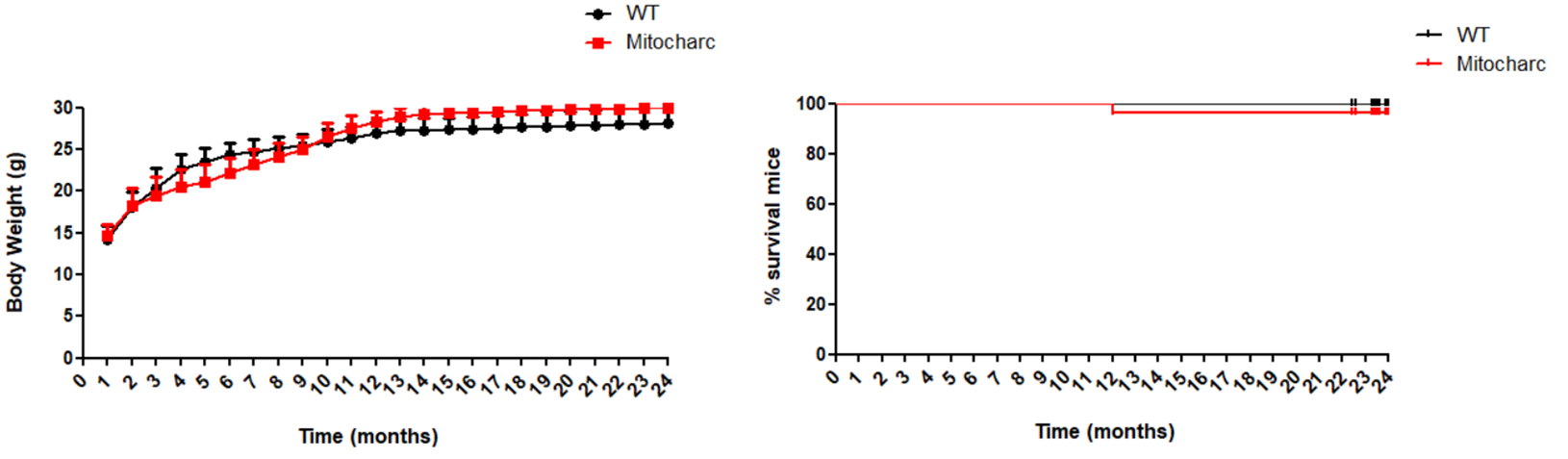


**Supplementary Fig. 5. MFN2 mice did not show reduced growth and survival.** **A** Body weight. Data are represented as mean ± SEM. n=26 for MFN2 and n=22 for WT mice per time point (1-24 months, months 12-24: *P<*0.001, Multiple t-test). **B** Kaplan-Meier survival curves for MFN2 (n=26) and WT (n=22). The animals were monitored from 1 to 24 months (*P=* 0,3576, Log-Rank test).

**
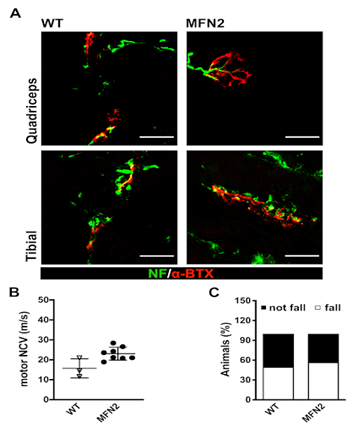
**

**Supplementary Fig. 6. MFN2 mice did not show motor phenotype alteration. A** Representative confocal image of α-bungarotoxin (α-BTX, red) and SMI32 (green) in quadriceps and tibial muscles from MFN2 *versus* WT mice (n=6/group, P270). Images were acquired using a Leica TCS SP5 confocal microscope (Leica Microsystems), at 20x magnification. Scale bar: 20 µm. **B** Quantification of motor nerve conduction velocity (NCV, m/s) of the tail nerves in WT (white triangles, n=3, P270) and MFN2 (black circles, n=8, P270, *P*=0,1152, Student’s t-test). **C** Rotarod test results of WT (n=10) and MFN2 (n=9) mice. The histogram showed the percentage of the mice that fall or not fall (*P*=0,3950, Student’s t-test).

**
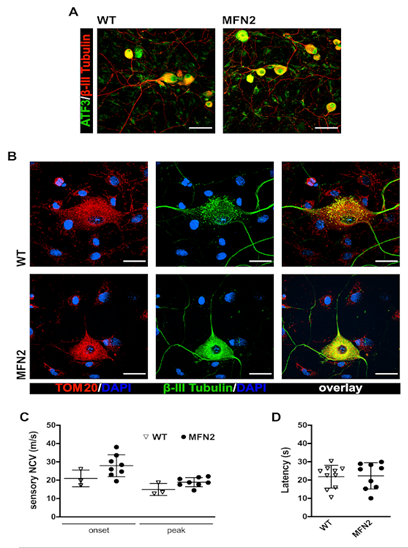
**

**Supplementary Fig. 7. MFN2 mice did not show neuro peripheral phenotype alteration. A** Representative image of WT and MFN2 mice (n=6/group) derived dorsal root ganglion (DRG) neurons stained with ATF3 (green) and βIII-Tubulin (red). Images were acquired using a Leica TCS SP5 confocal microscope (Leica Microsystems), at 40x magnification. Scale bar: 50 µm. **B** High-resolution confocal images of WT and MFN2 mice (n=6/group) derived DRG neurons labeled with TOM20 (red) and βIII-Tubulin (green). Nuclei were labeled with DAPI (blue). Images were acquired using a Video Confocal super-resolution (VCS) module on a Nikon ECLIPSE Ti/CREST microscope (Nikon), at 100x magnification. Scale bar: 25 µm. **C** Quantification of sensory NCS (m/s) of the tail nerves in WT (white triangles, n=3, P270) and MFN2 mice (black circles, n=8, P270) (Student’s t-test, onset: *P*= 0,1036, peak: *P*=0.0537). **D** Hot plate test results of WT (white triangles, n=10 P270) and MFN2 mice (black circles, n=9 P270). The graphic showed the latency (s) of the with drawal response of each hind paw during the test (Student’s t-test, *P*= 0,8934).

**
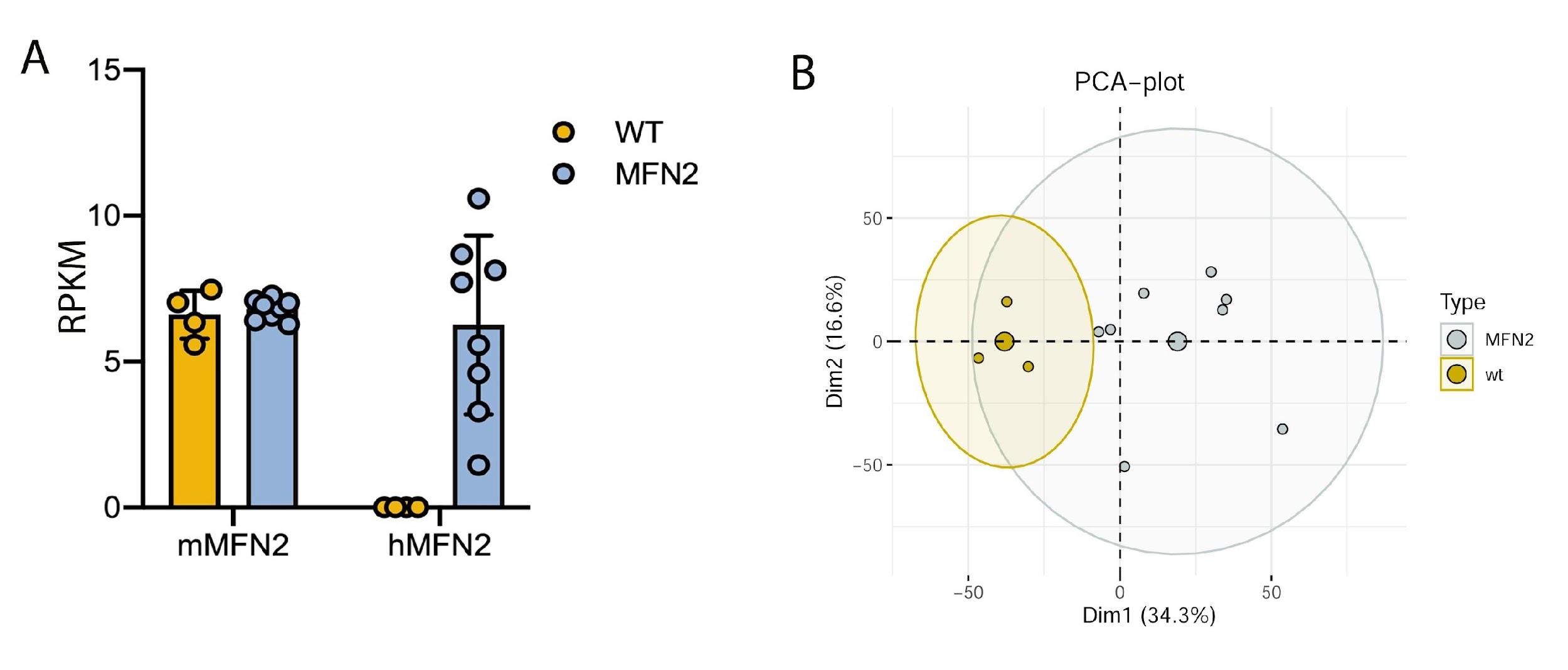
**

**Supplementary Fig. 8.** **A** RPKM of mMfn2 and hMFN2 reads obtained from RNA-Seq data of lumbar spinal cord (1-month-old) of MFN2 and WT animals. n = 4–8 mice/genotype (mMFN2: *P*= 0,5584; hMFN2: ***P<*0.01, Student’s t-test). **B** PCA of RNA-Seq data of lumbar spinal cord (1-month-old) n = 4–8 mice/genotype.

**References**

1. Leandri M, Saturno M, Cilli M, et al (2007) Compound action potential of sensory tail nerves in the rat. Exp Neurol 203:148–157. https://doi.org/10.1016/j.expneurol.2006.08.001

2. Nizzardo M, Simone C, Rizzo F, et al (2016) Morpholino-mediated SOD1 reduction ameliorates an amyotrophic lateral sclerosis disease phenotype. Sci Rep 6:21301. https://doi.org/10.1038/srep21301

3. Dobin A, Davis CA, Schlesinger F, et al (2013) STAR: ultrafast universal RNA-seq aligner. Bioinformatics 29:15–21. https://doi.org/10.1093/bioinformatics/bts635

4. Robinson MD, McCarthy DJ, Smyth GK (2010) edgeR: a Bioconductor package for differential expression analysis of digital gene expression data. Bioinformatics 26:139–140. https://doi.org/10.1093/bioinformatics/btp616

5. Subramanian A, Tamayo P, Mootha VK, et al (2005) Gene set enrichment analysis: a knowledge-based approach for interpreting genome-wide expression profiles. Proc Natl Acad Sci U S A 102:15545–15550. https://doi.org/10.1073/pnas.0506580102
